# Innovative Technologies for Articular Cartilage Repair: Research, Development, and Clinical Translation—A Narrative Review

**DOI:** 10.3390/jfb17030128

**Published:** 2026-03-05

**Authors:** Adriana Lorena Lara-Bertrand, Liliana Lizarazo-Fonseca, Luz Correa-Araujo, Gustavo Salguero, Ingrid Silva-Cote

**Affiliations:** Unidad de Ingeniería Tisular, Instituto Distrital de Ciencia Biotecnología e Innovación en Salud, Bogotá 111311, Colombia; alara@idcbis.org.co (A.L.L.-B.); alizarazo@idcbis.org.co (L.L.-F.); lscorrea@idcbis.org.co (L.C.-A.); gsalguero@idcbis.org.co (G.S.)

**Keywords:** articular cartilage, tissue engineering, biomaterials, regenerative medicine, osteoarthritis

## Abstract

Articular cartilage is a highly specialized connective tissue essential for joint function, providing load-bearing capacity, shock absorption, and near-frictionless motion. Due to its avascular nature, articular cartilage has a limited intrinsic healing capacity, and focal injuries often progress to degenerative joint diseases such as osteoarthritis, leading to chronic pain and functional impairment. This review examines current and emerging scientific, clinical, and technological strategies for articular cartilage repair and regeneration, with particular emphasis on their translational relevance. This narrative review integrates data from peer-reviewed literature, clinical trial registries, and patent databases. Preclinical and clinical approaches are discussed, including orthobiologics, cell-based therapies, advanced biomaterials, and three-dimensional tissue-engineered scaffolds. Bibliometric and keyword network analyses are used to identify dominant research themes, technological trends, and emerging innovations. The findings reveal a clear paradigm shift from conventional surgical interventions, often associated with fibrocartilage formation and suboptimal biomechanical performance, to multifactorial regenerative strategies combining cells, bioactive signals, and biomimetic scaffolds designed to recapitulate the native extracellular matrix. This convergence of regenerative medicine, tissue engineering, and biomaterials science is reflected in growing clinical translation efforts and intellectual property activity. Overall, although articular cartilage repair remains a significant clinical challenge, integrated regenerative approaches show great potential for achieving durable and functional cartilage regeneration.

## 1. Introduction

Cartilage is a strong and flexible connective tissue that covers the bone surfaces within joints. It is distributed across multiple anatomical locations, including articular joints, the rib cage, intervertebral discs, ears, and nose [1]. Functionally, cartilage provides structural support, absorbs mechanical shocks, lubricates joint surfaces, and enables smooth bone movement by reducing friction and wear. Three types of cartilage exist: hyaline, elastic, and fibrocartilage, each with distinct structural and functional characteristics [2].

Hyaline cartilage is composed of type II collagen and proteoglycans. It provides mechanical support and flexibility in structures such as the nasal septum and contributes to costal stability at the rib–sternum junction, while also maintaining airway patency in the trachea and larynx [3]. When present on joint surfaces, hyaline cartilage is referred to as articular cartilage, where it functions as a shock absorber and reduces friction between articulating bones. Articular cartilage is essential for joint function, acting as a load-bearing, low-friction surface that enables locomotion. It is avascular and aneural, with limited self-repair capacity; consequently, restoring or promoting endogenous repair of articular cartilage remains a significant clinical challenge [4].

With aging, joint cartilage gradually deteriorates, causing joint pain and swelling that are often only relieved by surgery. Articular cartilage injuries rarely heal spontaneously and usually progress to osteoarthritis (OA), the most common form of arthritis, which is characterized by chronic pain, stiffness, and progressive loss of joint function [2]. OA imposes a substantial burden on individuals and healthcare systems. Cartilage damage commonly results from sports-related trauma or mechanical injury. Elevated levels of inflammatory mediators, metabolic dysregulation, cellular senescence, and abnormal mechanical loading are key contributors to OA pathogenesis, contributing to surface erosion, extracellular matrix degradation, and persistent release of pro-inflammatory cytokines during the chondrocyte repair response [5]. Despite advances in understanding the molecular and biomechanical mechanisms underlying osteoarthritis, there is currently no therapy capable of restoring native hyaline cartilage structure and long-term joint function [6].

Despite significant advances in orthopedic surgery, treating cartilage damage remains a major clinical challenge. Current pharmacological therapies for OA, including monoclonal antibodies, acetaminophen, sprifermin (recombinant human fibroblast growth factor-18), and nonsteroidal anti-inflammatory drugs (NSAIDs), provide symptomatic relief but do not restore cartilage homeostasis [7]. Furthermore, conventional surgical and pharmacological approaches have several limitations, including graft instability, calcification, and limited applicability to focal cartilage lesions. These limitations have accelerated the development of new regenerative strategies based on cell therapy, gene modulation, and tissue engineering [8]. In addition to limited clinical efficacy, these approaches face challenges related to tissue integration, repair durability, scalability, and regulatory approval [9].

Tissue engineering is a promising interdisciplinary approach that integrates engineering principles with biological sciences to develop functional tissue substitutes capable of restoring or replacing damaged cartilage [5]. These strategies typically integrate cells, bioactive molecules, and scaffolding biomaterials to promote tissue regeneration. Three-dimensional (3D) scaffolds and hydrogels fabricated from natural or synthetic polymers, bioresorbable inorganic materials, hybrid composites, or decellularized tissues closely replicate the extracellular matrix, providing mechanical support and guiding the formation of functional neo-tissues. Concurrently, advances in biomaterial-based scaffolds, stem cell–based therapies, gene-modulated strategies, and bioactive medical devices have substantially broadened and strengthened the current therapeutic landscape [10,11]. Increasingly, effective cartilage regeneration is being pursued through multifactorial strategies that integrate cells, biomaterials, and bioactive cues rather than single-component solutions [8].

Clinically, articular cartilage repair remains one of the most difficult challenges in orthopedics due to its limited intrinsic healing potential [12]. Conventional techniques such as microfracture, autologous chondrocyte implantation (ACI), and osteochondral autografts or allografts are widely used but often result in incomplete regeneration or fibrocartilage formation with inferior biomechanical properties [13]. In response to these limitations, emerging regenerative strategies are increasingly being evaluated in clinical trials to assess their safety, efficacy, and translational potential [14]. Ultimately, successful clinical translation depends on bridging experimental innovation with patient outcomes through the integration of surgical techniques, regenerative medicine, and advanced biomaterials [15].

This review provides a comprehensive overview of the main scientific and technological strategies for articular cartilage repair. It encompasses preclinical approaches, including in vitro and animal studies, as well as the clinical application of orthobiologics, cell-based therapies, biomaterials, and tissue-engineered scaffolds. In addition, it examines relevant patents, clinical trials, and surgical procedures to offer an updated perspective on emerging technologies with translational and clinical potential. By integrating scientific evidence with clinical and technological perspectives, this review aims to identify emerging trends, translational opportunities, and remaining gaps to guide future research and development.

## 2. Materials and Methods

This study was designed as a narrative review aimed at evaluating emerging scientific, clinical, and technological strategies for the repair and regeneration of articular cartilage. Multiple complementary data sources were integrated to capture the research, clinical development, and innovation landscape of the field.

### 2.1. Data Sources

Scientific literature was collected from PubMed, Scopus, and Web of Science to identify peer-reviewed articles, reviews, and book chapters related to articular cartilage repair and regeneration. Clinical data were obtained from ClinicalTrials.gov and EudraCT, focusing on registered clinical studies addressing cartilage injuries and associated therapeutic interventions. Patent data were collected from Espacenet and PatBase, which provided global coverage of intellectual property activity in the field.

### 2.2. Inclusion and Exclusion Criteria

Studies published within the last ten years were prioritized to capture recent advances in articular cartilage repair and regeneration. Eligible documents included (i) scientific articles reporting in vitro or in vivo experiments, (ii) clinical trials evaluating cartilage repair interventions in humans, and (iii) patents describing biomaterials, devices, or methods relevant to articular cartilage regeneration. Duplicate records, studies unrelated to cartilage repair, and documents lacking sufficient technical or scientific relevance were excluded.

### 2.3. Data Extraction and Classification

For each selected document, the following variables were extracted: title, abstract, publication year, country of origin, treatment, technique, and study outcomes where available. For patents, additional information was recorded, including the family number, jurisdiction, legal status (active/inactive), the Cooperative Patent Classification (CPC) or International Patent Classification (IPC) codes, assignees, and inventors [16]. Clinical trials were classified by status (completed, recruiting, active but not recruiting, suspended, terminated, or withdrawn), treatment, and technique.

### 2.4. Analytical Approach

Although the document selection prioritized publications from the last ten years, technology monitoring and graphical analyses of scientific publications, clinical trials, and patents considered trends spanning approximately eighteen years. This broader temporal scope enabled the identification of long-term dynamics, emerging trajectories, and key turning points in scientific, clinical, and technological activity. The analysis included frequency assessments, temporal evolution, and the geographical distribution of publications, clinical trials, and patent families. Graphical representations were generated to visualize patent activity by publication year, legal status, and assignee. In addition, the analysis focused on interpreting emerging trends, identifying key technological domains, and examining interactions between scientific evidence, clinical translation, and intellectual property strategies. A strategic matrix was subsequently developed to assess opportunities and challenges associated with different technological approaches.

### 2.5. Keyword Network Analysis

A preliminary search was conducted to illustrate the field’s conceptual structure, and a technology map was generated using VOSviewer (version 1.6.20). This visualization displays the co-occurrence network of keywords extracted from the corpus of scientific publications, clinical studies, and patents. The size of the nodes represents the frequency of the keywords, and their colors reflect the thematic groups associated with tissue engineering, biomaterials, regenerative medicine, and scaffold manufacturing. In this map, colors indicate distinct thematic clusters identified through keyword co-occurrence. The orange cluster relates mainly to biomaterials and scaffold chemistry, the red cluster to animal and experimental studies, and the pink cluster to tissue engineering and clinical applications in humans. Together, these groups illustrate the conceptual structure and research focus areas within the field. The spatial distribution highlights conceptual proximities and emerging research fronts within the field.

**Figure 1:**
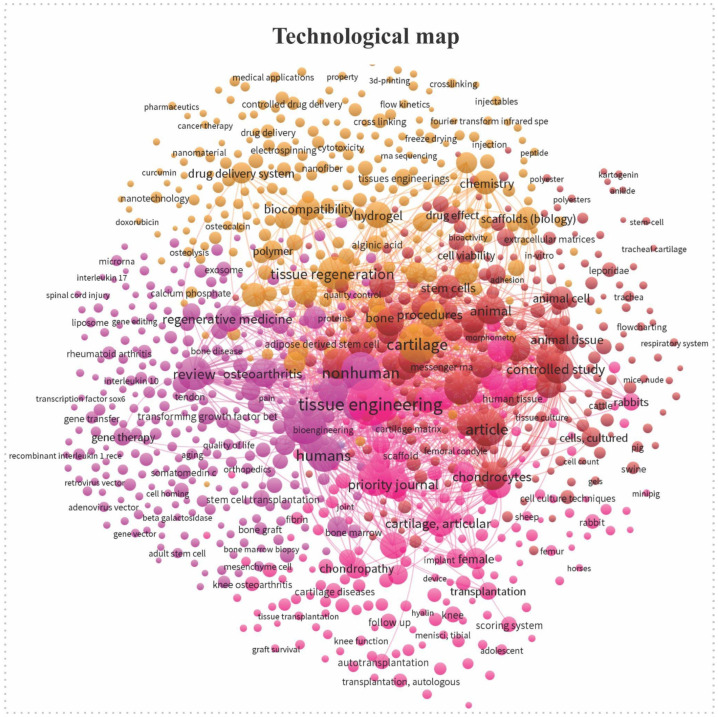
Technological map of research into osteochondral repair of articular cartilage generated from a co-occurrence network of keywords extracted from scientific literature.

The most frequent keywords identified were: “cartilage repair”, “cartilage tissue engineering”, “preclinical evaluation of cartilage repair”, “biomaterials”, “orthobiologics”, “exosomes in cartilage repair”, and “scaffolds” (Figure 1).

All documents written in English were included in the final analysis. Records in other languages, incomplete datasets, and conference proceedings were excluded. For clinical trials, the inclusion criteria were limited to studies involving human subjects with articular cartilage lesions, osteochondral injuries, or OA.

### 2.6. Integrated Technology Monitoring in Joint Cartilage Repair

The technological surveillance conducted in this review integrates data from scientific publications, patents, and clinical trials to provide an integrated overview of the current landscape of articular cartilage repair and regeneration. By combining these complementary sources, the approach captures the continuum of innovation, from basic research reported in peer-reviewed literature to translational activity reflected in clinical studies and technology protection strategies identified through patent families. This integrated perspective enables the identification of emerging trends, active research areas, existing limitations, and opportunities for future development, offering a strategic view of how knowledge generation, clinical validation, and technological innovation interact in cartilage regeneration.

Based on these objectives, an exhaustive search was carried out on the selected platforms, and the equation designed to carry out a specific technology watch of interest is presented below:

(cartilag* OR Articular cartilag* OR Knee cartilag* OR shoulder cartilag*) AND (meniscus OR condyle* OR patella OR femoral head OR rotator cuff) AND (device* OR system* OR mechanism* OR equipment*) AND (repair* OR reparation OR fix* OR regenera* OR restora* OR heal* OR cure) AND (tissue engineering) AND (treatment OR proces* OR therap* OR procedure*).

After conducting a comprehensive search using the previously defined search equation, the initial retrieval yielded a large amount of information from the three sources mentioned above for the scope of this literature review. Specifically, the search identified and compiled the following: 1225 peer-reviewed scientific publications, 230 relevant patents, and 353 clinical studies.

These identified records, totaling 1808 entries, were subsequently analyzed through a structured process of relevance assessment and data extraction to ensure that the information incorporated into this review was aligned with its scientific, clinical, and technological objectives. This approach may be influenced by the availability and completeness of databases and by the selective nature inherent to narrative reviews.

## 3. Results

### 3.1. Overview of Scientific Output, Reviews, and Book Chapters

**Figure 2:**
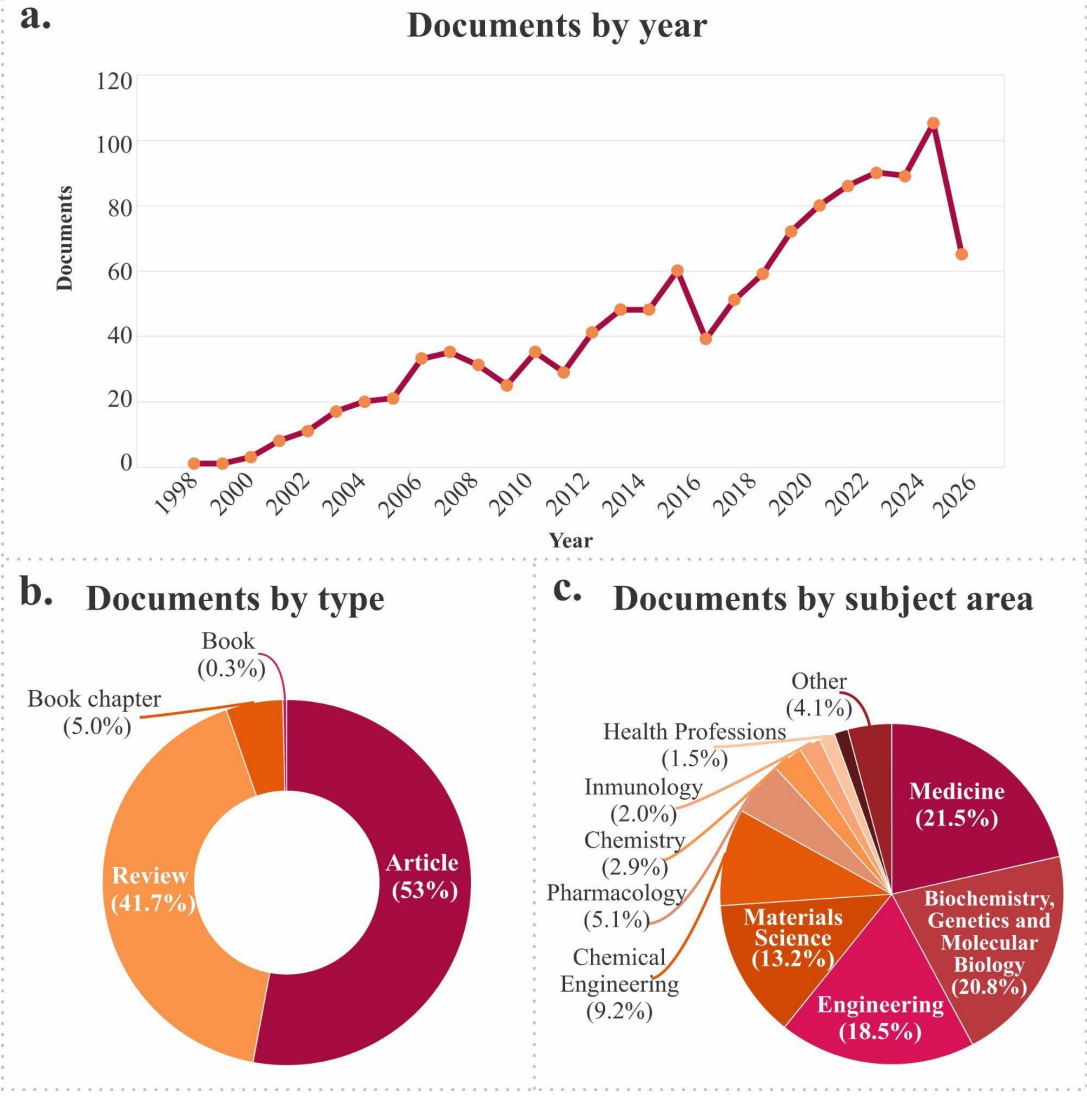
Bibliometric analysis of scientific literature related to articular cartilage repair. (**a**) Annual evolution of publications. (**b**) Distribution of document types. (**c**) Classification by subject area.

A search of scientific articles indicates a steadily increasing trend from 1998 to 2024, with a notable peak between 2008 and 2020, coinciding with the rise of emerging technologies such as advanced biomaterials, tissue engineering, 3D printing, and personalized cell therapies. This dynamic suggests strong research momentum to address joint pathologies through regenerative approaches. However, between 2021 and 2025, a slight decrease in production is observed, possibly attributable to a redistribution of scientific focus toward other global priorities or to the consolidation of established lines of research (Figure 2a).

Regarding publication type, 53% are original scientific articles. In comparison, 41.7% are systematic reviews or narratives, indicating that the scientific community is not only active in generating new knowledge but also committed to the critical synthesis and dissemination of existing information. The remaining percentage comprises book chapters, monographs, and other documents, indicating academic interest beyond the strict research field (Figure 2b).

Analysis of the predominant thematic areas reveals clear interdisciplinarity, with strong representation of clinical medicine (21.5%), molecular biology and genetics (20.8%), engineering (18.5%), and materials science (13.2%). This diversity reflects the fact that cartilage research lies at the intersection of basic, applied, and clinical sciences, enabling technological developments with direct relevance to public health. Taken together, the analyzed data confirm not only quantitative growth in literature but also the strategic relevance of this field within regenerative medicine and contemporary biomedical innovation (Figure 2c).

### 3.2. Clinical Evidence and Status of Trials in Cartilage

**Figure 3:**
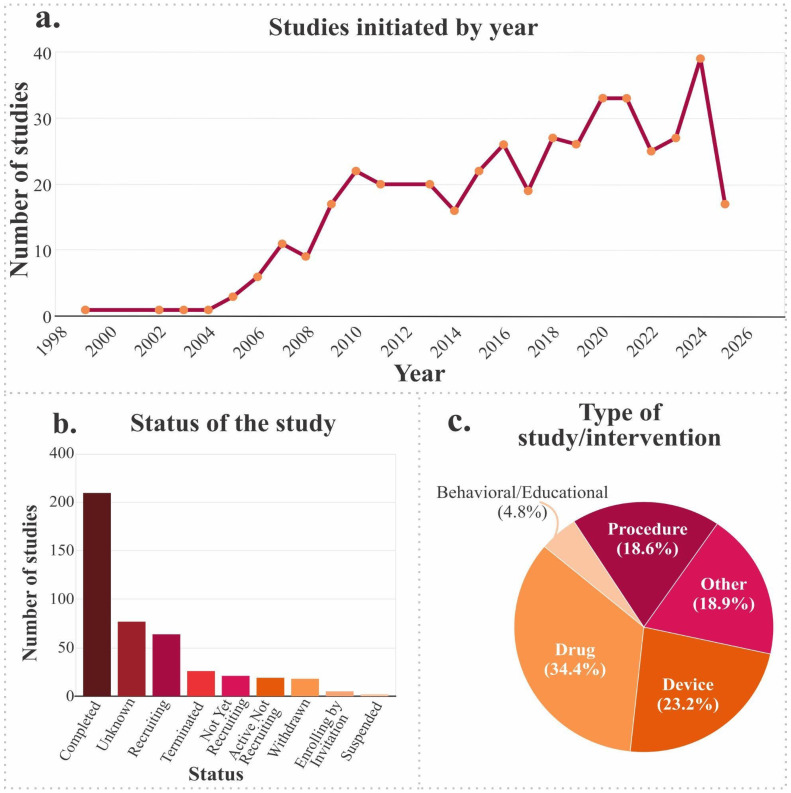
Clinical trial activity related to osteochondral cartilage repair. (**a**) Studies initiated per year. (**b**) Study status. (**c**) Type of study or intervention.

The identified clinical studies were analyzed to evaluate their temporal behavior and status. Figure 3a, which illustrates the distribution of documents by year, demonstrates a discernible upward trend from 1998 to 2024, with sustained growth accelerating particularly from 2010 onward. This pattern reflects an increasing interest among the scientific community in the study and treatment of cartilage, possibly motivated by recent advances in biotechnology, regenerative medicine, and the development of new biomaterials.

As illustrated in Figure 3b, which categorizes clinical studies by status, a considerable proportion are designated “completed,” indicating that a substantial number of investigational technologies have reached a sufficient level of maturity to be evaluated in humans. However, there is also evidence of numerous active studies, either in recruitment or in preparation, which demonstrate research dynamics in constant development.

The presence of studies that were terminated prematurely, suspended, or withdrawn may be associated with factors such as logistical difficulties, lack of promising outcomes, or the redistribution of resources toward more competitive research lines. An examination of the temporal progression of studies initiated per year reveals a gradual increase until approximately 2016, followed by a period of relative stabilization. This trend may reflect shifts in funding priorities, thematic saturation, or the emergence of more effective technologies that reduce the need for numerous exploratory trials.

Regarding the type of intervention recorded (Figure 3c), a significant proportion of studies (34.4%) focused on pharmaceuticals, while a notable proportion (23.2%) focused on medical devices, indicating a pronounced emphasis on applied therapeutic and technological solutions. A total of 18.9% of the interventions were classified as “other,” while 18.6% were classified as “procedures.” These categories may include combinations of surgical techniques or personalized methodologies. Educational or behavioral interventions represent only 4.8%, which is to be expected given the focus of this analysis on biomedical, pharmacological, and engineering approaches.

### 3.3. Overview of Technological Innovation and Intellectual Property Protection

The main objective of this review is to characterize the technological landscape for the repair and regeneration of articular cartilage, the sector’s level of activity, and the potential for development and innovation in therapeutic solutions.

As a result of this process, a total of 230 patent families were identified, spanning a wide range of approaches, from devices and equipment designed to facilitate the repair of damaged cartilage to tissue-engineering-based systems aimed at promoting the functional regeneration of cartilage.

Similarly, identifying technologies with inactive patents presents a strategic opportunity to develop innovations without legal restrictions, whereas analyzing active patents provides a clear view of the competitive environment, enables anticipation of potential barriers to market entry, and maps the current state of the field.

**Figure 4:**
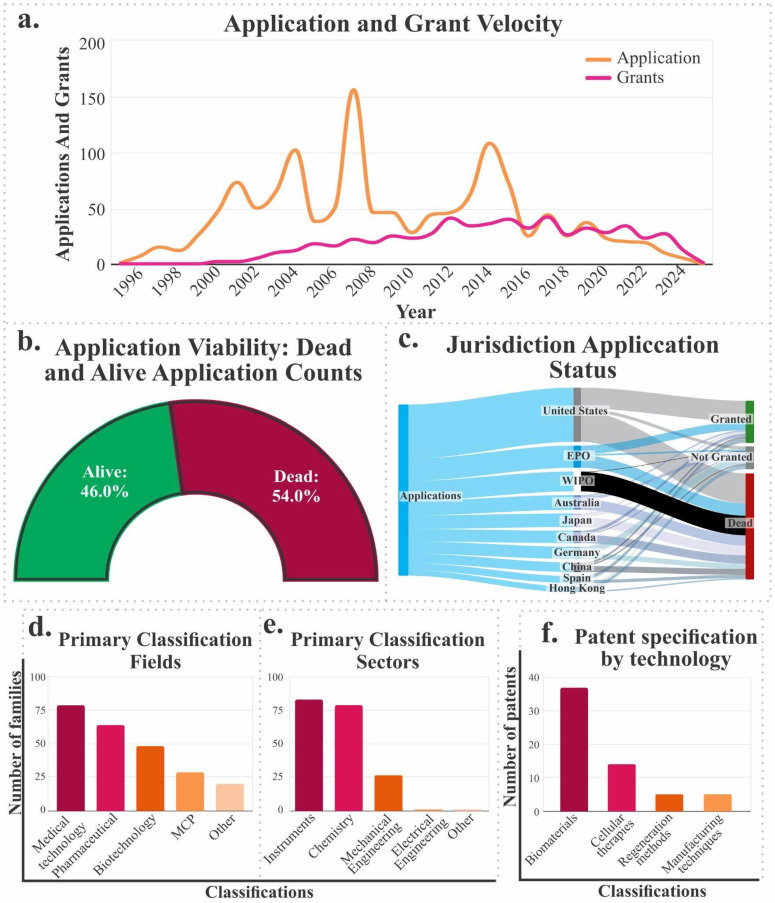
Analysis of patent documents. (**a**) Application and grant Velocity. (**b**) Application Viability. (**c**) Jurisdiction Application Status. (**d**) Primary Classification Fields. (**e**) Primary Classification Sectors. (**f**) Patent specification by technology.

Figure 4a shows the evolution of patent applications and grants between 1996 and 2024. A progressive increase in both indicators is observed, although applications consistently exceed grants, a predictable outcome given the duration of examination and approval processes. This trend reflects a competitive and innovation-driven environment in the development of cartilage-related technologies.

A complementary analysis reveals that approximately 54% of applications are classified as “inactive” (either due to abandonment, rejection, or non-continuation), while 46% remain active (Figure 4b), suggesting a high rate of technology leakage possibly associated with a lack of novelty, technical redundancy, or implementation feasibility issues.

When examining the geographical distribution of applications, the United States leads by a wide margin in terms of volume, followed by the European Patent Office (EPO), the World Intellectual Property Organization (WIPO), Australia, and Japan (Figure 4c). This distribution confirms that countries with greater scientific, regulatory, and intellectual property capacity are driving development in this field.

An analysis of technology classification codes reveals that most patents are concentrated in the medical, pharmaceutical, and biotechnology sectors, as well as in macromolecular chemistry polymers (MCP), indicating a strong focus on clinical solution development (Figure 4d). Contributions from chemistry, mechanical engineering, and electronic engineering are also identified, highlighting the multidisciplinary nature of biofunctional material device and interface development (Figure 4e).

It is important to note that technologies unrelated to cartilage may be identified during this review. For this reason, the main components of a patent (title, IPC, and CPC description) are analyzed. As a result, families were systematically classified and filtered to identify the most relevant ones, yielding 98 patent families directly related to the research area. These technologies were organized into five main categories, as shown in Figure 4f:

- Cell therapies: Involves the use of individual cells or cell microaggregates for tissue regeneration.
- Biomaterials: Includes polymers, hydrogels, fibers, and other advanced biomaterials used in joint cartilage repair.
- Regeneration methods: Groups together therapeutic strategies based on the application of growth factors and techniques aimed at stimulating cartilage repair.
- Manufacturing techniques: Includes advanced manufacturing technologies such as 3D printing, electrospinning, and other biofabrication methods applied to tissue scaffold design.
- Others: A set of technologies that, by their nature, do not fit directly into the above categories but are related to the subject of study.

Finally, the breakdown by specific technology type reveals a strong presence of inventions in cell therapies, advanced biomaterials, tissue regeneration strategies, and specialized manufacturing techniques. This reflects a convergence between biological and technological approaches to achieve functional restoration of articular cartilage.

### 3.4. Synthesis of Scientific, Clinical, and Patent Evidence from Orthobiologics to Biosynthetic Materials

Repairing articular cartilage is one of the most critical challenges in orthopedics and regenerative medicine. Technological innovation in cartilage repair has led to numerous patents, many of which focus on the design of biomaterials, multifunctional scaffolds, and hybrid regeneration strategies.

#### 3.4.1. Biological Strategies for Cartilage Engineering

In the context of cartilage repair, orthobiologics have emerged as advanced therapeutic strategies due to their capacity to modulate the inflammatory microenvironment and promote tissue regeneration, thereby reducing pain and accelerating healing while minimizing surgical-associated complications [17]. Currently available orthobiologic approaches include platelet-rich plasma (PRP), bone marrow aspirate concentrate (BMAC), micronized allogenic cartilage matrix (MCM), and mesenchymal stromal cell–based therapies. These biological modalities, encompassing cells, exosomes, and hemoderivatives, constitute a substantial component of current cartilage lesion management strategies, complementing conventional treatments such as pharmacological interventions, physical therapy, and surgical procedures [18]. Notably, extensive preclinical evaluation in animal models has demonstrated their regenerative potential, supporting their translational relevance and clinical applicability. The therapeutic outcome of these approaches is closely associated with lesion severity and the local tissue microenvironment.

Building upon this growing body of evidence, current translational efforts have increasingly focused on orthobiologic-based interventions as a cornerstone of contemporary cartilage repair strategies. These approaches aim to harness biological activity not only to stimulate intrinsic repair mechanisms but also to enhance the performance of biomaterial scaffolds and surgical techniques. Within this framework, cell-based therapies, exosome-mediated signaling, platelet-derived products such as PRP, and allograft and osteochondral systems have emerged as key therapeutic modalities. Each of these strategies addresses distinct biological and structural aspects of cartilage regeneration, ranging from immunomodulation and paracrine stimulation to the direct restoration of osteochondral architecture. The following sections examine these technologies in detail, highlighting their mechanisms of action, clinical indications, and current translational development status.

##### Cell Therapies

The advanced therapy medicinal products (ATMPs) constitute an emerging and innovative class of medicines used to treat multiple pathologies [19]. ATMPs include a diverse range of therapeutic products, such as gene therapy, tissue-engineered medicine, and somatic-cell therapy, which is among the most widely evaluated therapies for the treatment of articular cartilage.

Autologous mesenchymal stromal cells (MSCs) have emerged as a central biological strategy for cartilage regeneration, primarily due to the limited intrinsic repair capacity of articular cartilage. This tissue is composed of sparsely distributed chondrocytes embedded within a dense, avascular extracellular matrix, which severely restricts nutrient diffusion and cellular recruitment following injury. Mechanical and metabolic support is instead provided by the underlying subchondral bone, which plays a critical role in endogenous repair mechanisms. Clinical interventions such as microfracture techniques exploit this interaction by inducing the release of undifferentiated MSCs from the bone marrow into the defect site, thereby initiating a reparative response [20]. Beyond their capacity for differentiation, MSCs contribute to tissue regeneration through paracrine signaling and immunomodulatory effects, thereby further supporting their therapeutic relevance. Consequently, a wide range of regenerative approaches have focused on the direct administration of autologous MSCs for the treatment of articular cartilage lesions and degenerative joint diseases [21].

The translational potential of these strategies is supported by a growing body of clinical evidence. A comprehensive safety analysis of autologous MSC-based therapies for arthritis demonstrated an excellent safety profile, with no stem cell–specific adverse events and no increased risk compared with conventional arthritis treatments. These findings reinforce the clinical feasibility and safety of autologous MSC therapy for cartilage repair [22]. Although results like this reflect successful treatment and constitute an ATMP, a drawback of autologous MSCs is that the procedure requires two separate surgeries: one to harvest the patient’s own cartilage cells and another to implant the expanded cells into the defect. Moreover, this technique may require millions of cells to repair large defects, which, if left untreated, can cause symptoms such as pain, limit function, and may lead to osteoarthritis [23,24].

Allogeneic MSCs have also been extensively investigated for articular cartilage repair. A phase I (first-in-human) clinical trial evaluated the ability of allogeneic MSCs combined with either 10% or 20% recycled autologous cartilage-derived cells (chondrons) to promote cartilage regeneration in patients with symptomatic knee cartilage defects. All patients showed significant improvements in clinical outcomes compared with baseline after 12 months. In addition, magnetic resonance imaging and second-look arthroscopic assessments indicated complete filling of the defects with regenerated cartilage-like tissue [23]. Currently, multiple tissue sources of MSCs have been described, including dental pulp, periodontal ligament, tendon, skin, muscle, Wharton’s jelly, and other tissues. However, differences in isolation efficiency are strongly influenced by tissue availability, tissue condition, and donor age [25].

Autologous chondrocytes have been extensively applied in cartilage restoration strategies due to their inherent ability to contribute directly to hyaline cartilage repair. Their clinical use was first reported in 1994 by a Swedish research group, marking the introduction of ACI as a therapeutic approach for focal cartilage defects [26]. In this procedure, a small biopsy of healthy articular cartilage is harvested arthroscopically, typically from a non-weight-bearing region such as the proximal medial femoral condyle of the affected knee. Chondrocytes are subsequently isolated from the harvested tissue and expanded in vitro for 2–3 weeks to obtain a sufficient cell population (approximately 5 × 10^6^ cells) for implantation into the cartilage defect site [18,27].

From a technological and translational perspective, recent patent activity indicates a clear shift toward integrating biological components into engineered constructs. Multiple patent applications describe the incorporation of bone marrow– or adipose tissue–derived MSCs and cultured chondrocytes into biomaterial-based matrices or osteochondral grafts. This approach aims to convert traditionally passive implants into biologically active regenerative platforms that interact with the local tissue microenvironment.

In parallel, the incorporation of bioactive cues—such as growth factors including transforming growth factor beta (TGF-β), bone morphogenetic protein-2 (BMP-2), and insulin-like growth factor-1 (IGF-1)—is frequently reported, alongside anti-inflammatory or antimicrobial agents intended to mitigate post-surgical complications. Collectively, these design strategies illustrate the ongoing transition toward multifunctional therapeutic devices that not only replace damaged tissue but also actively modulate and direct the biological repair process [28].

Undoubtedly, ATMPs constitute a rapidly growing field in the treatment of a wide range of pathologies, with particular emphasis on cartilage restoration. However, these technologies face several challenges, including high costs and the need for specific administrative methods and specialized clinical expertise centers for administration and monitoring [29].

##### Exosomes

Exosomes are a subtype of extracellular vesicles (EVs) secreted by most cell types, with a characteristic size range of approximately 50–130 nm. These nanovesicles act as key mediators of intercellular communication by transporting a diverse cargo of bioactive molecules, including DNA, messenger RNA, microRNAs (miRNAs), and other non-coding RNAs such as long non-coding RNAs (lncRNAs) and circular RNAs (circRNAs), as well as proteins and lipids. Through this molecular cargo, exosomes regulate multiple biological processes, including immune modulation, tissue homeostasis, and coordinated cellular responses within complex microenvironments [30]. A variety of techniques are currently employed for the extraction, isolation, and purification of exosomes, each exploiting their specific physical and molecular properties. Commonly used methodologies include differential ultracentrifugation, density gradient centrifugation, kit-based precipitation methods, magnetic bead-based immunoaffinity capture, and size exclusion chromatography (SEC). However, the heterogeneity of isolation approaches remains a critical challenge for standardization and clinical translation [31].

Owing to their central role in cellular regulatory mechanisms, exosomes have been identified as efficient carriers of bioactive lipids, nucleic acids, and proteins directly involved in tissue regeneration. Increasing evidence supports their relevance in cartilage and bone repair, where exosomes have been shown to influence chondrogenesis, osteogenesis, and inflammatory responses. Consequently, they have been implicated in the development, diagnosis, and treatment of several orthopedic disorders, including rheumatoid arthritis (RA), OA, osteonecrosis, and osteoporosis, positioning exosomes as a promising cell-free therapeutic strategy [7,31,32,33].

The immunomodulatory potential of exosomes and microparticles (MPs) derived from bone marrow MSCs has been demonstrated in preclinical arthritis models. In BALB/c mice, exosomes and MPs isolated by differential ultracentrifugation were evaluated both in vitro and in vivo using delayed-type hypersensitivity (DTH) and collagen-induced arthritis (CIA) models. Treatment with these vesicles resulted in inhibition of T lymphocyte proliferation, accompanied by a reduction in CD4^+^ and CD8^+^ T-cell subsets. In the DTH model, MPs and exosomes exhibited dose-dependent anti-inflammatory effects, whereas in the CIA model, exosome administration significantly reduced clinical signs of inflammation. These therapeutic effects were further associated with decreased plasmablast populations and increased Breg-like cell populations in lymph nodes [33]. Beyond immunomodulation, exosomes have demonstrated regenerative efficacy in large-animal models. In a porcine osteochondral defect model, intra-articular administration of human MSC-derived exosomes combined with hyaluronic acid (HA) resulted in superior osteochondral repair compared with HA alone. Magnetic resonance imaging performed at 15 days, 2 months, and 4 months post-surgery revealed consistent cartilage formation and enhanced subchondral bone regeneration characterized by increased bone volume and trabecular thickness [34].

**Figure 5:**
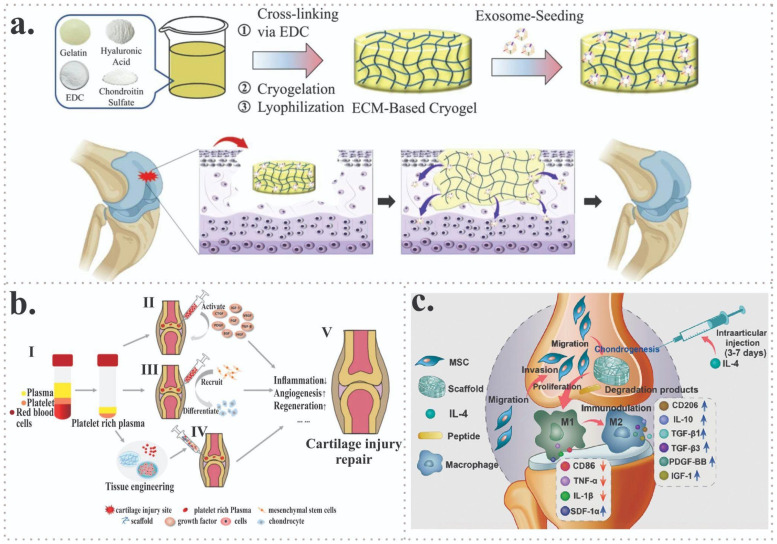
Biological therapeutic approaches evaluated in the restoration of cartilage injury. (**a**) Evaluation of exosome-seeded cryogel scaffolds [35]. (**b**) Role of PRP in the treatment of articular cartilage [36]. (**c**) Use of Cell-free decellularized cartilage extracellular matrix scaffolds combined with interleukin 4 [37].

More recently, biomaterial-based strategies incorporating exosomes have been explored to enhance their therapeutic performance. Exosome-seeded cryogels designed to mimic the extracellular matrix (ECM) have been fabricated via cryopolymerization using gelatin, chondroitin sulfate, and varying concentrations of HA, and subsequently enriched with human umbilical cord–derived MSCs (hUC-MSCs) exosomes (Figure 5a). When implanted into cartilage defects in New Zealand rabbits, both free exosomes and exosome-loaded cryogels promoted chondrocyte proliferation. Notably, cryogels—particularly those loaded with exosomes—significantly enhanced ECM deposition and sulfated glycosaminoglycan synthesis, highlighting the potential of ECM-mimetic, exosome-functionalized scaffolds for advanced cartilage tissue engineering applications [35].

##### Platelet-Rich Plasma (PRP)

PRP is an autologous blood-derived product obtained from peripheral blood and processed to selectively concentrate platelets, which are subsequently resuspended in plasma. Standard PRP preparation protocols typically involve centrifugation of whole blood at controlled speeds (commonly around 1500 g for 10 min), resulting in the separation into three distinct fractions: plasma (≈55%), platelet (≈1%), and red blood cells (≈45%) [15]. However, researchers have different opinions on which method to prepare PRP is more efficient and due to the great heterogeneity between PRP separation methods, its preparation method must be further standardized [36,38].

Undeniably, PRP exhibits a significantly higher platelet concentration and serves as a rich reservoir of bioactive molecules, including multiple growth factors implicated in tissue repair and regeneration (Figure 5b) [36].

The regenerative potential of PRP in cartilage repair has been extensively evaluated using rabbit models, which are well established for the study of osteochondral healing [39]. The therapeutic efficacy of PRP gel has been evaluated in a rabbit model of bilateral full-thickness cartilage defects created in the femoral trochlear groove. PRP-treated animals exhibited the formation of hyaline cartilage–like tissue at 12 weeks post-surgery, whereas control defects predominantly developed fibrocartilaginous or fibrous tissue. Notably, despite these long-term improvements, incomplete cartilage regeneration was observed in the PRP-treated group during the early postoperative period (3 weeks), highlighting a temporal dependence of PRP-mediated repair [40].

Beyond its use as a standalone therapy, the incorporation of PRP into biomaterial-based constructs has demonstrated synergistic effects on osteochondral regeneration. In a rabbit osteochondral defect model, the addition of autologous PRP to bilayer poly(lactide-co-glycolide) (PLGA) scaffolds significantly enhanced repair outcomes compared with PLGA scaffolds alone and untreated controls. Histological and macroscopic analyses revealed superior tissue organization in the PLGA/PRP group, accompanied by increased expression of cartilage-specific markers, such as type II collagen and aggrecan. Furthermore, micro-computed tomography (micro-CT) imaging demonstrated enhanced subchondral bone formation in PRP-functionalized scaffolds, underscoring the osteoinductive and chondrogenic synergy between PRP and engineered biomaterials [41,42].

In order to investigate the effects of PRP on regeneration capacity as well as survival of the cartilage grafts, a study was carried out in a rabbit model. In this case, auricular cartilage was used to obtain 4 pieces of cartilage (each about 2 × 2 cm) and PRP was prepared using 5 mL of auricular blood. Cartilage pieces were then mixed with either normal saline or PRP and then implanted on the back of the rabbit. Histological analysis showed that grafts enriched with PRP resulted in increasing regeneration of chondrocytes, accompanied by angiogenesis, and diameters of vessels increased after 12 weeks post treatment [43].

Although PRP has shown significant advances in the repair of articular cartilage injuries, there are still some controversies regarding its mechanism of action, its effect on long-term clinical treatment, and its preparation method, due to its complex composition [36].

##### Allografts and Osteochondral Systems

The repair of articular cartilage following injury remains a significant clinical challenge due to its limited intrinsic regenerative capacity. In this context, decellularized tissues have emerged as a promising therapeutic strategy, providing bioactive scaffolds capable of supporting tissue repair and remodeling. Decellularized extracellular matrix (dECM) derived materials have been extensively explored for cartilage restoration, as they retain the native biochemical composition of the tissue, including structural proteins such as collagen, as well as endogenous growth factors and cytokines that promote cell viability, proliferation, and matrix deposition [10]. dECM can be generated from a wide range of tissues using diverse decellularization and processing methodologies, including bone, cartilage, meniscus, tendon, skin, adipose tissue, urinary bladder, small intestinal submucosa, liver, and brain [10,43,44,45,46,47]. This versatility has enabled the development of tissue-specific scaffolds with preserved architectural and biochemical cues. In a representative in vivo study, cartilage repair was achieved using acellular osteochondral allografts engineered to preserve surface lubrication properties. Osteochondral grafts harvested from donor sheep were decellularized, lyophilized, and stored prior to implantation into critical-sized femoral condyle defects. After six months, acellular allografts demonstrated superior integrative cartilage repair compared with untreated defects, while maintaining native-like structural organization, biochemical composition, and tribological performance. Notably, high cellular infiltration was observed within the bone compartment and at the cartilage graft interface, confirming effective integration and long-term biomechanical functionality [48].

Another study evaluated the regenerative potential of porcine decellularized cartilage matrix (DCM) combined with interleukin 4 (IL-4) in an osteochondral defect model. Standardized osteochondral defects were created in the femoropatellar groove of Sprague-Dawley rats and treated with DCM scaffolds, DCM combined with IL-4, or left untreated as controls.Histological analysis at 8 weeks demonstrated that the DCM scaffold loaded with 10 ng of IL-4 achieved the most pronounced osteochondral regeneration, characterized by improved cartilage morphology and subchondral bone restoration. Both the DCM alone and the DCM + 50 ng IL-4 groups also showed superior repair outcomes compared with the untreated controls. These findings indicate that DCM scaffolds combined with precisely tuned immunomodulatory cues can significantly enhance osteochondral regeneration, highlighting immuno-informed biomaterial design as a promising strategy for cartilage repair (Figure 5c) [37].

From a translational and technological perspective, document analyses related to osteochondral allografts and autologous transplantation techniques, including osteochondral autograft transfer systems (OATS), mosaicplasty, and fresh osteochondral allografts (OCAs), indicate that these approaches remain the clinical gold standard for the treatment of extensive cartilage defects or in cases where less invasive interventions have failed. For fresh allografts, recent inventions emphasize advanced cell-preservation protocols, surface functionalization strategies, and bioactive coatings designed to enhance graft integration with host bone while prolonging chondrocyte viability. Additionally, modular graft designs have been proposed to improve adaptability to defects with complex or irregular geometries, thereby reducing surgical time and optimizing fixation [49].

Although osteochondral autografts offer superior biocompatibility, their clinical application is constrained by donor-site morbidity and limited tissue availability. To address these limitations, hybrid strategies have been developed in which small autograft plugs are combined with filler biomaterials, such as hydrogels or biodegradable polymers, effectively reducing the volume of harvested tissue while maintaining repair efficacy [50].

An emerging and particularly promising direction involves the integration of bioprinted scaffolds with osteochondral grafts, combining the immediate mechanical stability of native tissue with the regenerative and customizable potential of next-generation biomaterials. This hybrid approach is especially attractive for the treatment of complex osteochondral lesions in young patients, where long-term joint preservation and functional recovery are critical considerations [51,52].

#### 3.4.2. Advanced Biomaterials for Cartilage Engineering

Biosynthetic scaffolds have become a central platform in regenerative medicine, particularly for cartilage repair, by providing a 3D microenvironment that not only supports cell adhesion, proliferation, and differentiation but also actively regulates tissue regeneration. Engineered to recapitulate key structural, mechanical, and biochemical features of the native ECM, these scaffolds function as temporary templates that guide neotissue formation while undergoing controlled degradation in synchrony with tissue maturation [53].

Within this framework, advanced biomaterials for cartilage engineering increasingly rely on biofabrication strategies, defined as the automated and spatially controlled assembly of biomaterials, cells, and bioactive molecules to generate biologically functional constructs. Importantly, biofabrication is an umbrella term encompassing multiple enabling technologies, including microfluidics, electrospinning, and 3D printing, rather than being synonymous with any single technique. This distinction is critical, as 3D printing constitutes a subset of biofabrication rather than an independent or parallel concept, in accordance with the standardized terminology proposed by Moroni et al. [54].

Recent advances in biofabrication technologies, including microfluidics, electrospinning, and 3D bioprinting, have markedly increased control over scaffold architecture, enabling precise modulation of pore size, fiber alignment, spatial cell distribution, and biochemical functionalization. These technological developments have substantially expanded the translational relevance of scaffold-based strategies for OA therapy and focal cartilage repair. However, despite these technological advances, most studies continue to evaluate individual scaffold designs in isolation, with limited direct comparison between competing material classes or fabrication strategies, making it difficult to define performance benchmarks or identify clear advantages across approaches.

Among the design parameters governing clinical performance, biocompatibility and biodegradability remain fundamental. Biocompatibility ensures safe interaction with host tissues by minimizing cytotoxicity, immunogenicity, and adverse inflammatory responses, whereas biodegradability enables progressive scaffold resorption and its replacement by newly formed tissue, thereby promoting seamless structural and functional integration [55]. Nonetheless, long-term validation of these properties under physiological loading conditions remains inconsistent, and negative outcomes such as premature mechanical failure or fibrocartilaginous repair are rarely reported or systematically analyzed.

Current approaches in cartilage tissue engineering can be broadly categorized based on material composition and biological complexity, including naturally derived biomaterials, synthetic polymeric systems, hybrid constructs incorporating cells, and advanced nanocomposite biomaterials fabricated through biofabrication platforms. Each strategy offers distinct advantages and limitations in terms of bioactivity, structural support, and translational potential. Despite this diversity, comparative studies directly evaluating these strategies under standardized experimental conditions remain scarce, limiting critical assessment of reproducibility, robustness, and clinical relevance.

##### Natural Biomaterials: Collagen and Hyaluronic Acid

Both natural and synthetic biomaterials have been extensively investigated to meet the complex biological and mechanical requirements of osteochondral tissue engineering. Among naturally derived biomaterials, collagen and HA are particularly attractive due to their intrinsic bioactivity, close resemblance to the native ECM, and ability to support chondrogenic differentiation. Collagen, as the primary structural protein of cartilage ECM, provides mechanical integrity and essential cell-adhesion cues, whereas HA contributes to the viscoelastic properties of the tissue and plays a key role in regulating cell migration, inflammatory responses, and matrix turnover [56].

The composition of collagen and HA-based scaffolds critically influences their mechanical performance, including stiffness and tensile strength. Increasing the relative content of these biomolecules has been shown to enhance the elastic modulus, thereby improving the ability of the scaffold to approximate the mechanical behavior of native cartilage. In parallel, scaffold porosity is a decisive parameter governing nutrient diffusion, waste removal, and cellular infiltration. However, porosity must be carefully optimized, as excessive porosity compromises mechanical stability, whereas insufficient porosity can impede tissue integration and regeneration [57]. While these design principles are well established, most studies focus on optimizing individual parameters rather than systematically comparing competing scaffold formulations, limiting insight into trade-offs between mechanical performance and biological functionality.

Beyond composition and porosity, scaffold geometry, particularly at the macroscale, should be tailored to the anatomical and biomechanical characteristics of the target joint [58]. Cylindrical scaffold geometries, for example, have proven advantageous for applications in intervertebral discs and specific regions of the knee, as they facilitate uniform load distribution and help preserve joint congruency under physiological loading conditions [59]. At the microscale, studies using electrospun gelatin fibers with controlled diameters have demonstrated that matrix stiffness strongly modulates chondrocyte biosynthetic activity, underscoring the importance of precise mechanical tuning in scaffold design [60]. However, the reproducibility of these finely tuned architectures across fabrication platforms and laboratories is rarely addressed, raising concerns regarding scalability and standardization.

In addition to structural optimization, biofunctionalization strategies have been widely adopted to actively guide cellular behavior. The incorporation of growth factors such as TGF-β and BMP-2 enables targeted promotion of chondrogenic and osteogenic differentiation, respectively. When delivered through scaffold-based controlled-release systems, these signaling molecules allow for spatiotemporal regulation of tissue formation, enhancing the coordination of cartilage and subchondral bone regeneration [61]. Despite promising biological outcomes, few studies critically evaluate dose-dependent effects, long-term stability, or potential adverse responses associated with sustained factor release.

The long-term translational potential of natural biomaterial-based scaffolds has been demonstrated in large-animal studies. A comprehensive 15-year in vivo evaluation using an ovine model, selected for its close anatomical and biomechanical similarity to the human knee joint, showed that cell-seeded natural biomaterial scaffolds composed primarily of collagen- and hyaluronic acid–based matrices supported autologous chondrocyte survival, extracellular matrix deposition, and durable hyaline-like cartilage regeneration within full-thickness defects. Importantly, excellent tissue integration, mechanical functionality, and the absence of immune rejection were maintained throughout the extended follow-up period, highlighting the robustness and clinical relevance of this approach [62].

Complementary evidence has been provided by long-term evaluations of biomaterial-mediated repair of focal cartilage defects using hybrid scaffolds combining natural and synthetic components in an ovine model. These engineered constructs, integrating collagen-based matrices with biodegradable polymers, were designed to replicate the native cartilage microenvironment while providing enhanced mechanical support. Histological and biomechanical analyses revealed stable formation of hyaline-like cartilage, effective integration with host tissue, and restoration of joint function without significant adverse immune responses. Collectively, these findings reinforce the promise of biomaterial-based scaffold strategies as viable long-term solutions for cartilage regeneration and their potential translation into clinical therapies [63]. While these studies provide rare long-term validation, their limited number and heterogeneity in design highlight the need for broader comparative and multicenter investigations to establish clinical robustness.

##### Nanocomposite and Piezoelectric Biomaterials

In recent years, nanocomposite biomaterials have gained increasing attention as advanced scaffold systems capable of integrating mechanical reinforcement, bioactivity, and dynamic responsiveness. By incorporating nanoscale fillers such as carbon nanotubes, graphene derivatives, bioactive nanoceramics, or polymeric nanofibers into polymeric or hydrogel matrices, nanocomposite scaffolds can more closely replicate the hierarchical organization and functional complexity of native cartilage ECM [64,65].

Among these platforms, piezoelectric nanocomposite hydrogels represent a particularly promising and rapidly evolving class of functional biomaterials for cartilage regeneration. These systems incorporate piezoelectric components—such as barium titanate (BaTiO_3_), zinc oxide (ZnO), or poly(vinylidene fluoride) (PVDF)-based nanostructures—within compliant matrices, enabling the conversion of physiological mechanical loading into localized electrical cues. Recent studies have demonstrated that this mechanoelectrical stimulation promotes mesenchymal stem cell chondrogenic differentiation, enhances glycosaminoglycan and type II collagen deposition, and modulates inflammatory signaling without requiring external electrical stimulation [66,67,68]. In this context, the electrically active microenvironment mimics the endogenous bioelectromechanical signals present in native cartilage during joint motion.

In parallel with nanoparticle-reinforced systems, intrinsically piezoelectric hydrogels, based on electroactive polymer networks without inorganic nanofillers, have recently emerged as a distinct strategy for cartilage repair. These materials generate electrical signals in response to mechanical deformation or ultrasound stimulation while maintaining structural homogeneity and injectability. Recent in vivo studies have shown that such hydrogels can recruit endogenous mesenchymal stem cells, enhance TGF-β–mediated chondrogenic differentiation, and promote the formation of hyaline-like cartilage in osteochondral defect models. In addition to stimulating extracellular matrix deposition, these systems have been shown to modulate the inflammatory microenvironment associated with osteoarthritis, underscoring their potential as cell-free, minimally invasive therapeutic platforms. From a translational perspective, the absence of inorganic nanoparticles may improve fabrication reproducibility, reduce long-term biosafety concerns, and facilitate regulatory classification compared with composite counterparts [69].

Compared with conventional hydrogels or mechanically reinforced nanocomposites, piezoelectric systems offer the unique advantage of dynamically interacting with the mechanically active joint environment, enabling on-demand bioelectrical stimulation synchronized with physiological loading. Nevertheless, most studies remain limited to short-term in vitro experiments or small-animal models, and direct benchmarking against clinically established scaffold-based therapies is still lacking. These limitations currently restrict the assessment of long-term functional stability, large-scale manufacturability, and safety, which are essential parameters for clinical translation.

##### Synthetic Biomaterials: Poly(ε-caprolactone) and Poly(lactic-co-glycolic acid)

Poly(ε-caprolactone) PCL and poly(lactic-co-glycolic acid) (PLGA) are among the most widely employed synthetic polymers in tissue engineering, owing to their excellent mechanical tunability, batch-to-batch reproducibility, and controllable degradation kinetics. PCL, characterized by its semicrystalline structure and slow degradation rate, provides prolonged mechanical stability, rendering it particularly suitable for load-bearing applications. Moreover, its surface chemistry can be readily modified or functionalized with bioactive molecules to enhance cell adhesion, proliferation, and lineage-specific differentiation. Recent studies have demonstrated that hybrid PCL-based scaffolds, when combined with synovial-derived MSCs and tetrahedral framework nucleic acids (tFNAs), significantly promote chondrogenesis and ECM synthesis, underscoring the versatility of PCL as a bioactive scaffold platform [70,71].

Similarly, PLGA, which is composed of lactic and glycolic acid monomers, offers a highly tunable degradation profile that can be precisely adjusted by modulating the lactic-to-glycolic acid ratio. This tunability enables synchronization between scaffold resorption and neotissue formation, while also supporting localized delivery of growth factors or therapeutic agents. These attributes make PLGA particularly attractive for osteochondral scaffold design, where coordinated regeneration of articular cartilage and subchondral bone is essential [72]. Despite their widespread use, comparative evaluations against natural biomaterials under identical loading and biological conditions remain limited, complicating objective assessment of long-term functional superiority.

For scaffolds intended for load-bearing tissues such as articular cartilage, both mechanical and structural properties must be carefully engineered. Key design parameters, including material composition, porosity, pore interconnectivity, and macrogeometry, collectively dictate mechanical performance, degradation behavior, and biological integration. Proper optimization of these factors ensures not only sufficient structural support but also the establishment of a permissive microenvironment that facilitates cell infiltration, nutrient diffusion, and extracellular matrix deposition [73].

Analysis of the current patent landscape reveals that a substantial proportion of osteochondral scaffold technologies rely on biodegradable synthetic polymers such as polylactic acid (PLA), PCL, and their copolymers. These materials are favored for their biocompatibility, adjustable mechanical properties, and compatibility with advanced manufacturing techniques, including 3D printing. To enhance bioactivity and integration at the osteochondral interface, many designs incorporate bioceramic components such as hydroxyapatite or calcium carbonate. In parallel, multiphasic and gradient scaffolds have emerged as a key design strategy to recapitulate the structural and functional transition between cartilage and underlying bone, thereby addressing the limitations of homogeneous biomaterials, which often result in fibrocartilaginous repair tissue [74]. However, patent-driven innovation frequently emphasizes material novelty over systematic reporting of failure modes, reproducibility, or cost-effectiveness.

Collectively, these findings highlight the translational potential of synthetic polymer–based and hybrid biomaterial scaffolds for cartilage repair. Their mechanical robustness, design versatility, and demonstrated efficacy in relevant preclinical models support continued development toward clinically applicable strategies for the treatment of cartilage injuries and osteochondral defects [75].

##### Combined Biomaterials and Cells

Surface nanotopography and micropatterning constitute powerful strategies to modulate cell adhesion, morphology, and lineage commitment by recapitulating key architectural features of the ECM. Engineered surface cues—such as microgrooves, nanopillars, and aligned fibrous architectures—have been shown to regulate cytoskeletal organization and mechanotransduction pathways, ultimately influencing gene expression and promoting tissue-specific cellular responses. In parallel, MSCs have emerged as key functional components for scaffold bioactivation, owing to their dual capacity for lineage differentiation and paracrine signaling. Under appropriate biochemical and mechanical stimuli, MSCs can differentiate toward chondrogenic phenotypes while simultaneously secreting cytokines and growth factors that modulate inflammation, support angiogenesis, and facilitate ECM remodeling. The incorporation of MSCs within scaffold matrices has therefore been shown to enhance biological integration and contribute to the mechanical maturation of regenerated cartilage tissue [76,77].

In this context, a biomimetic three-dimensional scaffold for cartilage repair was developed using a hybrid fabrication strategy that integrates natural and synthetic biomaterials, specifically collagen and poly(ε-caprolactone) (PCL). This composite design was intended to balance biocompatibility, mechanical integrity, and controlled biodegradation while closely replicating the hierarchical organization of the native cartilage extracellular matrix. Scaffold performance was evaluated through comprehensive in vitro and in vivo studies, including implantation into full-thickness cartilage defects in a rabbit model. The results demonstrated robust chondrocyte proliferation, enhanced extracellular matrix deposition, and the formation of hyaline-like cartilage characterized by organized type II collagen expression and appropriate matrix architecture. Functional assessments further revealed improved joint mobility and reduced cartilage degeneration in scaffold-treated animals compared with controls [75].

Although these findings support translational potential, the predominance of small-animal models and relatively short follow-up periods continues to limit their predictive value for human clinical outcomes. While cell-laden and biofabricated scaffolds consistently show superior biological outcomes, their increased complexity introduces challenges related to manufacturing scalability, reproducibility, regulatory approval, and cost, which are rarely addressed in preclinical studies. The development of next-generation cartilage scaffolds, therefore, relies on the rational integration of biomaterials, cells, and biofabrication technologies, rather than on isolated optimization of individual components.

The development of next-generation scaffolds for cartilage regeneration relies on the synergistic integration of biocompatibility, biodegradability, mechanical robustness, and bioactivity. By strategically combining ECM-derived natural biomaterials with the structural versatility of synthetic polymers and further integrating bioactive cues and stem cell–based therapies, multifunctional scaffolds can be engineered to closely recapitulate the native joint microenvironment. These advances are driving the transition toward personalized and effective regenerative strategies for osteoarthritis and other degenerative joint disorders. Moving forward, key challenges include rigorous in vivo validation, scalable manufacturing processes, and regulatory-compliant clinical translation. In parallel, recent patent activity highlights growing interest in injectable, cell-laden hydrogel systems incorporating MSCs, chondrocytes, growth factors, or bioactive peptides. Such minimally invasive formulations conform to irregular defect geometries and enable in situ tissue regeneration, thereby expanding the therapeutic landscape for cartilage repair [78]. Nevertheless, comparative analyses against acellular systems are limited, and few studies critically evaluate whether the added biological complexity justifies the increased translational burden.

##### Biofabrication Strategies and In Situ Bioprinting Approaches

Biomanufacturing encompasses automated, spatially controlled technologies for assembling cells, biomaterials, and bioactive molecules into functional biological constructs. Within this framework, 3D bioprinting is a subset of biofabrication techniques that enables the precise deposition of biological inks according to predefined architectures. In contrast, conventional 3D printing refers to the fabrication of scaffolds that lack biological components.

Among biofabrication-based approaches, 3D bioprinting, particularly in situ bioprinting, has emerged as a clinically relevant strategy for cartilage repair, allowing direct fabrication of constructs within the defect site and ensuring optimal conformity to complex geometries and improved tissue integration. Recent advances demonstrate the translational potential of in situ bioprinting for cartilage regeneration. Robotic-assisted in situ bioprinting systems have been developed to deposit cell-laden bioinks with high spatial accuracy, promoting cartilage matrix formation and defect filling under clinically relevant conditions [79]. Handheld and 4D bioprinting approaches further expand this concept by enabling surgeon-controlled deposition of smart, stimuli-responsive bioinks directly within cartilage defects, offering promising avenues for minimally invasive repair [80]. Earlier foundational studies demonstrated the feasibility of handheld in situ bioprinting for cartilage regeneration, establishing the basis for subsequent technological advancements [81].

A critical comparison of these studies highlights complementary strengths and distinct limitations. Robotic-assisted in situ bioprinting systems offer superior spatial precision, reproducibility, and automated control over deposition parameters, making them particularly attractive for standardized defect filling and complex geometries under preclinical or controlled clinical settings [79]. In contrast, handheld and 4D in situ bioprinting approaches prioritize surgical flexibility and real-time adaptability, enabling surgeon-guided deposition directly within irregular cartilage defects and supporting minimally invasive workflows [80,81]. However, these handheld systems may exhibit greater operator-dependent variability and lower architectural precision than robotic platforms, potentially affecting reproducibility and scalability.

Overall, the integration of advanced biomaterials with biofabrication strategies, particularly in situ bioprinting, highlights a clinically oriented approach that bridges material design, manufacturing precision, and surgical applicability in cartilage tissue engineering.

The convergence of advanced biomaterials with biofabrication technologies is further reflected in emerging patent trends, in which three-dimensional (3D) bioprinting is increasingly integrated with controlled delivery systems for bioactive agents, giving rise to smart, biofabricated scaffolds capable of dynamically modulating the joint microenvironment [82]. Despite these promising developments, variability in biofabrication protocols and material formulations remains a major challenge for reproducibility and regulatory translation.

**Figure 6:**
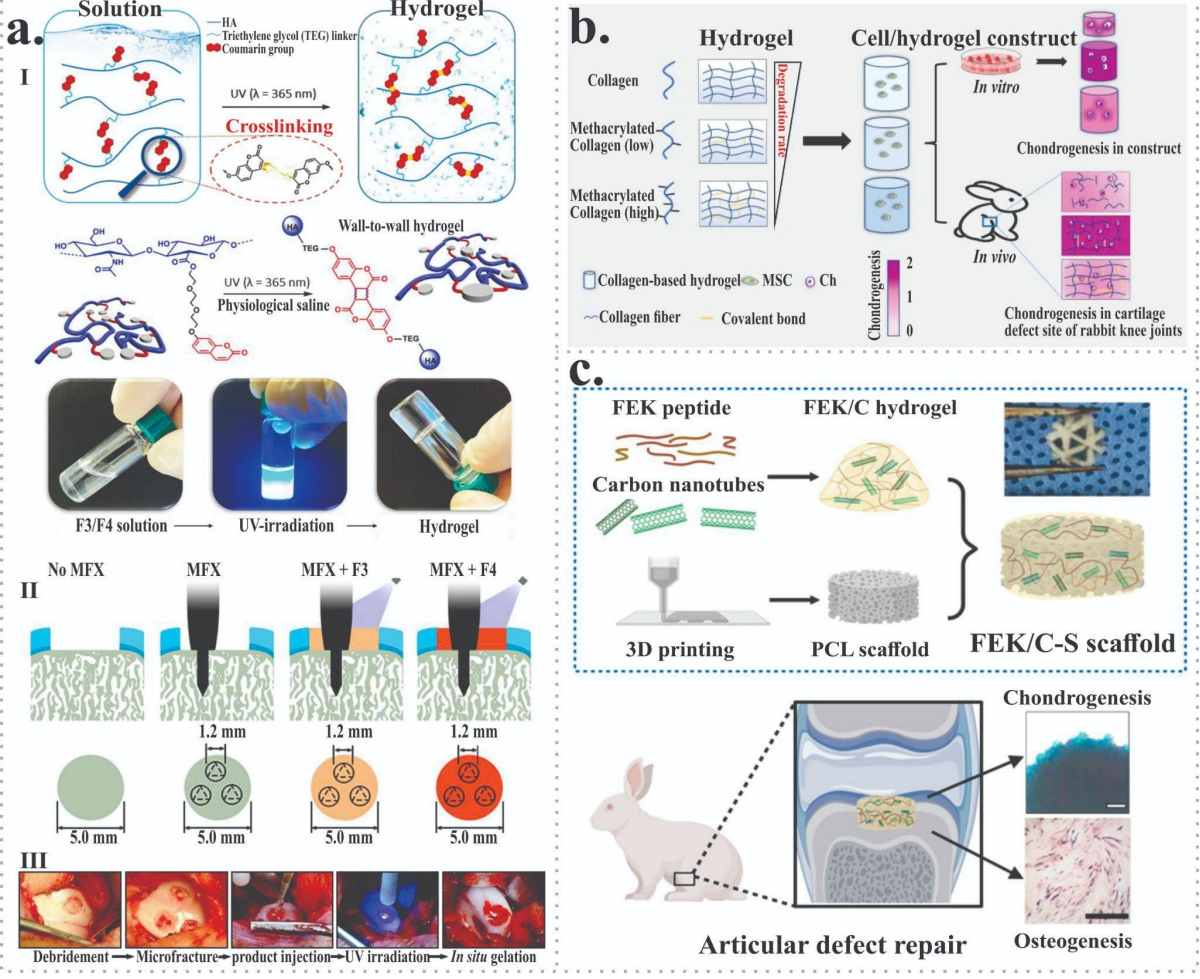
Schematic overview of representative hydrogel-based strategies for cartilage and osteochondral regeneration reported in recent studies: (**a**) a photopolymerizable, biocompatible hyaluronic acid–based hydrogel that promotes early articular cartilage repair in a minipig in vivo model [85]. (**b**) collagen-based hydrogels with controlled degradation rates that regulate mesenchymal stem cell chondrogenic differentiation and enhance cartilage regeneration [64] and (**c**) carbon nanotube–doped peptide hydrogel–polycaprolactone composite scaffolds that improve cartilage and subchondral bone repair through optimized scaffold properties [65].

In this context, bioactive 3D-printed scaffolds used in combination with microfracture have been shown to significantly enhance cartilage regeneration in a rabbit model, promoting increased cartilage thickness, higher chondrocyte density, and elevated expression of type II collagen and glycosaminoglycans compared with microfracture alone (Figure 6a) [48,83,84,85]. Compared with in situ bioprinting approaches, this strategy offers higher manufacturing control and material reproducibility, but lacks the ability to adapt scaffold architecture intraoperatively to patient-specific defect geometries. Together with recent advances in biofabricated hydrogel and nanocomposite scaffold systems [86,87,88], these findings highlight the potential of biofabrication-driven strategies for clinically relevant cartilage regeneration, while underscoring the need for long-term and standardized validation. Recent investigations have further focused on advanced scaffold designs to enhance cartilage repair. Collagen-based hydrogels with tunable degradation rates have been shown to promote mesenchymal stromal cell chondrogenic differentiation and significantly improve cartilage regeneration both in vitro and in vivo, particularly when intermediate degradation profiles are used (Figure 6b) [64]. In addition, carbon nanotube–doped peptide hydrogel–poly(ε-caprolactone) composite scaffolds have demonstrated enhanced mechanical support, improved cell proliferation and differentiation, and effective repair of both cartilage and subchondral bone, underscoring the critical role of scaffold composition and architecture in osteochondral regeneration (Figure 6c) [65].

### 3.5. Integration of Scientific, Clinical, and Patent Evidence for Orthopedic Research

Following completion of individual analyses of scientific publications, clinical studies, and patent documents, data derived from the previously interpreted graphical outputs were systematically cross-referenced to identify which technologies have been successfully implemented in orthopedic clinical practice and the therapeutic and surgical strategies currently applied to patients. This integrative approach enabled a comprehensive articulation of the results by bridging fundamental research, tissue engineering innovations, and real-world clinical adoption.

The comparative assessment revealed convergent evolution across scientific research, clinical investigation, and technological development, although with varying degrees of maturity. The results show that sustained growth in preclinical and translational research has enabled partial progression toward clinical application; however, a substantial proportion of emerging technologies remains at early or intermediate stages of development. This imbalance reflects measurable gaps between technological innovation, availability of robust clinical evidence, and routine implementation in orthopedic practice.

**Figure 7:**
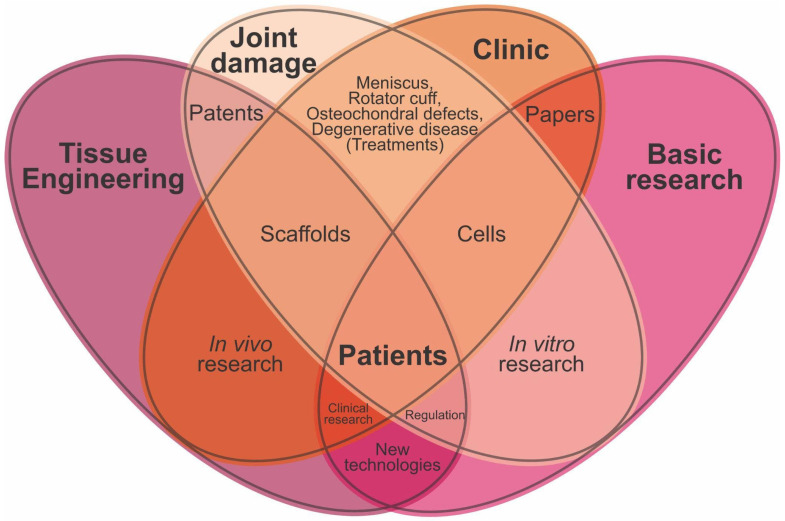
From basic research to clinical practice: Integrative framework showing the convergence of clinical practice, joint damage, tissue engineering, and basic research in the field of articular cartilage repair. Patients are placed at the center, where scaffolds, cells, patents, and publications intersect with in vitro, in vivo, and clinical research, together with regulatory and technological innovations.

Importantly, the cross-referencing strategy also enabled a critical evaluation of the advantages, limitations, and incremental advances of the therapeutic approaches currently used in patients, alongside the regulatory and translational barriers to their deployment. Key challenges include demonstrating long-term safety and efficacy, generating high-quality clinical evidence, and aligning with evolving regulatory frameworks. These interdependencies are summarized in Figure 7, which illustrates the dynamic interactions among joint pathology, clinical decision-making, basic research, and tissue engineering, positioning the patient at the center of the translational continuum and highlighting the outstanding challenges in consolidating effective, clinically scalable therapies for articular cartilage regeneration.

#### 3.5.1. Current Clinical Perspectives on Articular Cartilage Therapies: Surgery, Biomaterials and Biological Therapies

Throughout this review, not only preclinical findings and clinical trial data were considered, but a structured joint assessment was also performed to evaluate real-world clinical evidence and its practical applicability. The synthesis was organized according to therapeutic categories and surgical techniques, encompassing palliative interventions such as chondroplasty and debridement [89], repair strategies including drilling and microfracture [90] and tissue restoration approaches such as autologous chondrocyte implantation (ACI/MACI), autologous osteochondral transfer (OATS/mosaicoplasty), and osteochondral allografts [91]; Complementary procedures, including meniscal repair or transplantation and other adjuvant interventions, were considered when clinically indicated. This framework was aligned with established clinical evaluation models, including the IDEAL framework and AAOS guidelines, thereby enabling contextualization of the results with respect to clinical relevance and patient-specific scenarios.

**Figure 8:**
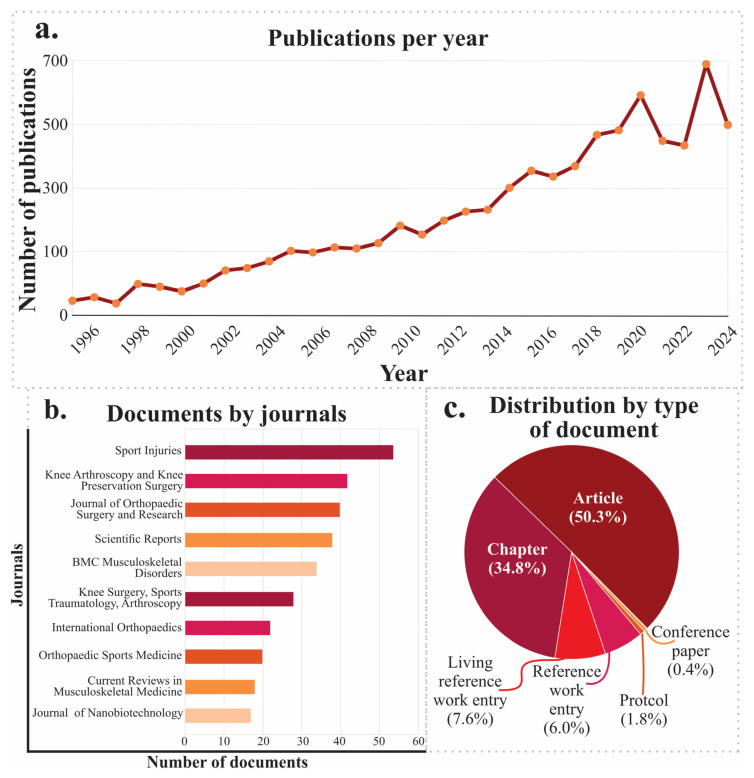
From basic research to clinical practice. (**a**) Publications per year. (**b**) document by Orthopedic Journals. (**c**) Distribution by type of document.

Scientific output on articular cartilage regeneration has shown a consistent upward trajectory from 1996 to 2024, with a pronounced acceleration after 2010 and a peak in publications in 2021, reflecting increasing research and clinical interest in this field (Figure 8a).

Analysis of publication venues reveals that many studies are concentrated in journals specializing in orthopedics and sports medicine, with Knee Surgery, Sports Traumatology, Arthroscopy, and Knee Surgery & Related Research standing out, followed by Journal of Orthopedic Surgery and Research, Scientific Reports, and BMC Musculoskeletal Disorders (Figure 8b). Regarding document types, original research articles predominate (50.3%), followed by book chapters (34.8%) and study protocols (18.6%), with a smaller contribution from conference proceedings and reference works (Figure 8c). This distribution not only highlights the growth in primary evidence generation but also reflects an increasing diversification of scientific dissemination formats within the field.

#### 3.5.2. Surgical Techniques Used in the Implementation of Technologies for the Repair of Articular Cartilage

Based on the comprehensive analysis of the available evidence, it can be concluded that articular cartilage degeneration results from the interplay of mechanical overload, traumatic injury, and chronic inflammatory processes. These factors contribute to the development of focal cartilage lesions and, in more advanced stages, to the progression toward osteoarthritis. Such pathological changes predominantly affect weight-bearing joints, particularly the knee, and, once symptomatic, often necessitate surgical intervention, including repair or regenerative strategies.

Within this therapeutic landscape, currently available approaches encompass bone marrow stimulation techniques, such as microfracture and microdrilling; regenerative strategies, including autologous chondrocyte implantation and bioengineered osteochondral scaffolds; and osseointegrative methods based on osteochondral grafting [92]. The selection of each technique is influenced by lesion size, anatomical location, patient age, activity level, and the condition of the surrounding joint environment. From a surgical standpoint, these procedures share critical steps, including meticulous defect debridement, preparation of stable vertical margins, and precise depth control to optimize implant stability and biological response.

In parallel, the incorporation of emerging technologies, such as three-dimensional biomaterial scaffolds, functionalized hydrogels, cell-based therapies, controlled-release systems, and extracellular matrix–derived constructs, requires careful integration with appropriate surgical techniques. This includes the selection of fixation methods (press-fit, sutures, bioadhesives), control of intraoperative bleeding, and postoperative load management. This alignment is critical for preserving implant viability, promoting stable integration with host tissues, and restoring the biomechanical and functional properties of the osteochondral unit. Together, these considerations underscore the need for a multidisciplinary, translational approach to advance effective, durable solutions for articular cartilage regeneration.

#### 3.5.3. Clinical Considerations

As outlined above, the selection of a cartilage repair procedure is primarily determined by lesion-related factors such as size, depth, location, and containment, as well as patient-specific variables including age, comorbidities, activity level, and functional demands. In parallel, the technological and regulatory maturity of the proposed intervention must be carefully evaluated, particularly when investigational products or advanced therapies are considered, since the availability of clinical evidence and regulatory approval status directly influence clinical decision-making.

From a practical standpoint, current interventions for articular cartilage repair can be categorized according to their biological target and surgical complexity, as summarized below.

##### Procedures Aimed at Bone Marrow Stimulation

- Microfracture and microperforation techniques: Bone marrow stimulation techniques, including microfracture and microperforation, are based on controlled penetration of the subchondral bone to induce bleeding and facilitate the migration of mesenchymal stromal cells into the cartilage defect. Surgically, these procedures begin with meticulous debridement of the lesion to achieve stable vertical margins and removal of the calcified cartilage layer. Subsequently, perforations are created using an awl or drill, typically spaced 3–4 mm apart and 3–4 mm deep, to access the bone marrow niche.
- Although microfracture remains one of the most widely performed cartilage repair techniques and is frequently considered a first-line surgical option, evidence consistently indicates that the resulting repair tissue predominantly consists of fibrocartilage, which is biomechanically and biochemically inferior to native hyaline cartilage [93]. Long-term follow-up studies report that, while short-term symptom improvement is common, structural deterioration and clinical failure may occur beyond five years postoperatively, largely independent of lesion size [94].
- However, from a surgical perspective, microfracture remains attractive due to its versatility, reproducibility, and feasibility via minimally invasive arthroscopic approaches.
- Microfracture combined with bioactive support: To overcome the limitations associated with fibrocartilage formation, microfracture has been combined with bioactive scaffolds, including hydrogels and porous membranes. These biomaterials stabilize the marrow-derived clot, enhance cell retention, and promote neochondrogenesis. This strategy has demonstrated improved outcomes in focal cartilage lesions and enables the incorporation of growth factors or other bioactive cues to further modulate tissue regeneration [95].

##### Osteochondral Grafts

- Mosaicplasty: Mosaicplasty involves harvesting cylindrical osteochondral plugs from low-load-bearing donor sites within the same joint and transplanting them into focal chondral or osteochondral defects. Recipient sockets are prepared using drills sized slightly larger than the graft length, allowing press-fit fixation. Multiple grafts are placed in a mosaic-like configuration to restore the articular surface.

Donor sites are typically left untreated and heal through fibrocartilage formation within 4–8 weeks, often supported by concomitant microfracture or microperforation [96]. While mosaicplasty offers the advantage of immediate hyaline cartilage transfer, its application is limited by donor-site morbidity and defect size.

- Osteochondral allograft: This technique eliminates donor-site morbidity and allows the treatment of larger or more complex defects. However, its clinical use is constrained by graft availability, high costs, and potential risks related to immunological response, graft resorption, and incomplete integration [97]. These procedures generally require more extensive surgical exposure and are most commonly performed through mini-open or open approaches rather than arthroscopy.
- Open surgery (mini-open or total approach): The implantation of rigid, multilayered, or patient-specific scaffolds, particularly those produced via additive manufacturing, often necessitates an open or mini-open surgical approach. This provides direct access to the defect site and facilitates precise positioning, fixation, and osseointegration of osteochondral or composite implants.

##### Tissue Engineering Techniques

- Autologous chondrocyte implantation (ACI): ACI is a two-stage procedure that begins with arthroscopic harvesting of a small full-thickness cartilage biopsy from a low-load-bearing region. Chondrocytes are isolated and expanded ex vivo, yielding approximately 12–48 million cells. During a second surgery, the cells are implanted into the prepared defect and covered with a periosteal flap or membrane.

The principal advantages of ACI include the use of autologous cells—minimizing immunological risks—and reduced donor-site morbidity compared with osteochondral autografting [98].

- Matrix-induced autologous chondrocyte implantation (MACI): MACI represents an evolution of ACI, in which autologous chondrocytes are seeded onto a biodegradable three-dimensional scaffold (e.g., collagen or HA–based matrices) prior to implantation. Fixation is achieved using sutures or biological adhesives. This approach improves cell distribution, simplifies implantation, and has led to the development of clinically approved products, such as MACI^®^.
- Autologous matrix-induced chondrogenesis (AMIC): AMIC is a single-step procedure combining microfracture with the application of a collagen or polymeric membrane that stabilizes the marrow clot and supports chondrogenic differentiation. This approach bridges traditional marrow stimulation and scaffold-based regeneration.

Advanced Therapies and Combinations

- MSCs, platelet-rich plasma, biomaterials, and gene therapy: Emerging strategies increasingly explore multimodal combinations involving MSCs, PRP, bioactive scaffolds, and gene therapies. While each modality has shown promise individually, their combined application remains largely investigational, with limited standardized clinical protocols and evolving safety frameworks. Nevertheless, such integrative approaches may offer more durable regenerative outcomes [8].
- Arthroscopic implantation: Arthroscopy remains the most widely adopted minimally invasive approach for treating chondral and osteochondral lesions. It enables the delivery of injectable hydrogels, microspheres, exosome-loaded formulations, and flexible or self-adapting scaffolds. This approach is particularly advantageous for thermo- or photo-responsive biomaterials that polymerize in situ with minimal tissue disruption [99].
- Image-guided intra-articular injection: Injectable therapies—including MSC suspensions, PRP, purified exosomes, and controlled-release nanoplatforms—are commonly administered under imaging guidance in outpatient settings. These approaches are typically used in early-stage joint degeneration or as adjuvant therapies to stimulate endogenous repair mechanisms.

Clinical evidence indicates that PRP plays a role not only in coagulation but also in modulating inflammation, angiogenesis, and ECM synthesis through the release of growth factors [100]. Randomized clinical trials have demonstrated that intra-articular PRP injections significantly reduce pain and improve function in knee osteoarthritis, with superior outcomes compared with prolotherapy or saline injections over follow-up periods of 6–12 months, particularly in patients with early-stage disease [101,102].

#### 3.5.4. Surgical Management and Translational Maturity of Cartilage Repair Technologies

In patients presenting with symptomatic cartilage damage, surgical decision-making must integrate defect characteristics, patient expectations, functional demands, and prior interventions. Cross-analysis of surgical application modes and development stages demonstrates that translational maturity is closely linked to surgical feasibility and procedural complexity. Techniques that can be performed arthroscopically, such as microfracture or injectable biological therapies, have achieved broader clinical adoption despite limited regenerative outcomes. Conversely, procedures requiring open or mini-open approaches, including ACI, MACI, and osteochondral grafting, exhibit higher levels of clinical evidence but are constrained by greater surgical complexity, higher costs, and greater rehabilitation demands. Advanced scaffold-based and biofabricated technologies often require non-standardized implantation strategies, which currently represent a significant barrier to widespread clinical translation [103,104].

Evidence-based management algorithms—derived from randomized controlled trials—provide guidance on selecting appropriate cartilage repair strategies [105]. Evaluation of the relevant actors in cartilage repair technologies further highlights stratification by developmental stage. Clinically established interventions, such as ACI and MACI, are predominantly driven by industrial stakeholders with regulatory approval and standardized manufacturing pipelines. Technologies at an intermediate stage of translation, including osteochondral grafting systems, are primarily supported by specialized hospitals, tissue banks, and clinical research centers. In contrast, early-stage biomaterial scaffolds, hydrogels, and biological augmentation strategies are largely developed within academic institutions and research-driven biotechnology enterprises, reflecting their exploratory status and the ongoing need for translational and regulatory consolidation [106,107].

Table 1 synthesizes the current landscape of cartilage repair by summarizing the principal technologies, biomaterials, biological approaches, and surgical techniques discussed in this review, integrating their translational maturity, clinical application modes, advantages, limitations, and levels of evidence. This comparative analysis reveals a clear gradient in clinical validation: established procedures such as ACI and MACI demonstrate the highest level of clinical maturity, supported by comparative studies and randomized controlled trials, whereas bone marrow stimulation techniques such as microfracture, despite their widespread use, exhibit variable evidence and limited long-term durability. In contrast, emerging biomaterial-based scaffolds, injectable hydrogels, and biological augmentation strategies remain largely at preclinical or early clinical stages, highlighting a persistent gap between technological innovation and robust clinical validation.

#### 3.5.5. Translational, Regulatory, and Clinical Challenges in Cartilage Repair Therapies

Despite major advances in cartilage repair technologies, including biological therapies, biomaterials, and tissue-engineered constructs, clinical translation remains limited by intertwined regulatory, manufacturing, and clinical barriers. Regulatory frameworks governing these products are often heterogeneous and insufficiently tailored to advanced regenerative therapies, generating uncertainty during development and approval processes [120,121].

Regulatory classification represents a primary challenge. Depending on source, manipulation, and intended use, cartilage repair strategies may be regulated as medical devices, biological products, ATMPs, or combination products. In the United States, minimally manipulated autologous or allogeneic Human Cells and Tissue-Based Products intended for homologous use may not require premarket approval under FDA 21 CFR Part 1271, although allogeneic products must meet donor eligibility and traceability requirements [122]. More complex combination products integrating cells, biomaterials, or bioactive components are subject to stricter regulatory pathways, increasing development time and cost [121,123].

Manufacturing scalability and economic feasibility further constrain translation. Advanced scaffold-based systems and cell-based therapies require GMP-compliant environments, leading to high production costs, batch variability, and challenges in quality control and long-term sustainability [123]. Clinical translation is also limited by the poor predictive value of current preclinical models. Existing animal models often fail to replicate human joint biomechanics, inflammatory conditions, and long-term functional demands, and standardized large-animal models and clinically predictive endpoints remain lacking. Clinical translation is also limited by the poor predictive value of current preclinical models. Existing animal models often fail to replicate human joint biomechanics, inflammatory conditions, and long-term functional demands, and standardized large-animal models and clinically predictive endpoints remain lacking. For example, Moran et al. (2016) mentioned disadvantages of various in vivo models commonly used in the assessment of biomaterial strategies for cartilage repair, such as very small joints in situ that make examination in mice impossible, increased cell density in cartilage in rats, different loading characteristics that influence results in rabbit trials, difficulty in achieving skeletal maturity in pigs, and subchondral cyst formation in goats [124]. Consequently, promising in vivo results frequently do not translate into durable clinical benefit, and long-term human data are scarce [125]. Preclinical approaches to translate cartilage products successfully require large-animal studies that meet regulatory requirements; this topic is a key step towards obtaining authorization for initiating human clinical trials [126].

Technology-specific challenges persist across different approaches. Synthetic biomaterials must reproduce the biomechanical and tribological properties of hyaline cartilage while ensuring long-term stability and tissue integration. Allografts and osteochondral allografts face additional barriers related to donor variability, cell viability, limited shelf life, and disease transmission risks, requiring stringent quality and traceability controls [127,128].

Across all strategies, compliance with international quality, biocompatibility, risk management, and clinical investigation standards is essential, although regional regulatory differences continue to complicate global translation [120].

Additional regulatory and clinical challenges are technology-specific. For synthetic biomaterials, key limitations include reproducing the biomechanical and tribological properties of native hyaline cartilage, ensuring long-term functional stability under joint loading, and achieving predictable Overall, effective clinical implementation of next-generation cartilage repair therapies will require early integration of regulatory planning, scalable manufacturing strategies, and clinically relevant preclinical models to bridge the gap between experimental success and sustainable clinical use [121].

## 4. Discussion

The search for therapeutic alternatives to promote articular cartilage repair remains a major challenge in orthopedic medicine, largely due to the unique physiology and anatomy of cartilage, particularly its limited intrinsic self-healing capacity [8]. Our search identified multiple treatment alternatives focused on cartilage restoration, drawing on in vitro and in vivo studies, related patents, and clinical translation, while considering regulatory aspects, clinical trials, and geographical perspectives.

The comparative analysis performed across multiple search platforms enabled the integration of scientific, technological, and clinical data from a geographical perspective. In terms of scientific output, China leads in the number of published documents, followed by the United States and Germany. Significant contributions were also identified from the United Kingdom, Italy, India, South Korea, Iran, Japan, and Switzerland, reflecting a broad international distribution of academic interest in cartilage repair. This global engagement underscores the diversity of approaches being explored, as well as a growing emphasis on achieving complete tissue restoration through individual or combined therapeutic strategies, each offering specific advantages depending on their biological and technological characteristics.

Regarding patent activity, evaluated by the number of patent families, the United States again demonstrates clear leadership, followed by the WIPO, the EPO, Canada, and Germany. This trend highlights the significant role of Anglo-American and European regions in the protection and commercialization of cartilage repair-related technological innovations.

**Figure 9:**
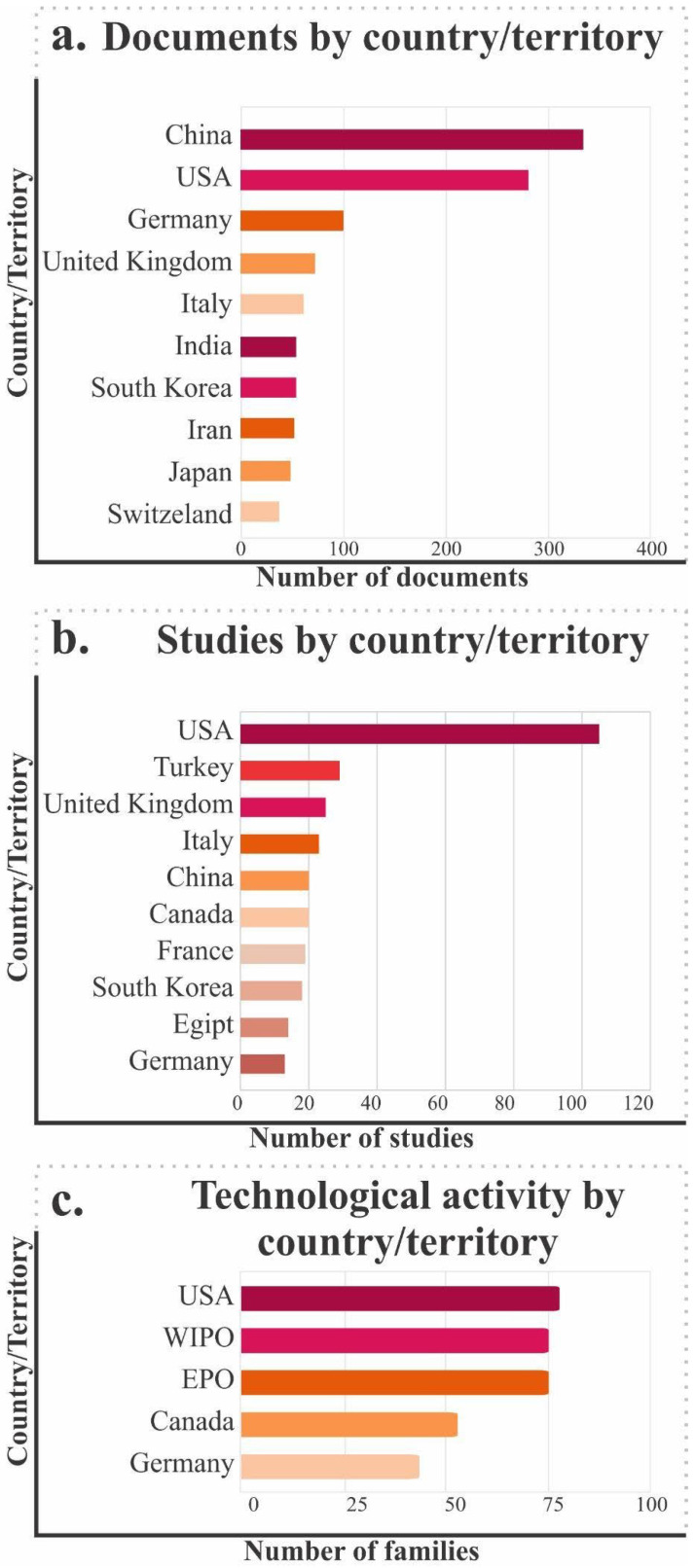
Analysis of database platforms by country. (**a**) Documents by country/territory (papers, books, or reviews). (**b**) Studies by country/territory (clinical trials). (**c**) Technological activity by country/territory (patent families).

In the clinical research landscape, the United States also ranks first in the number of active or completed clinical studies, followed by Turkey, the United Kingdom, Italy, and China. Notably, France, South Korea, Egypt, and Germany also show substantial participation. This distribution indicates that both established scientific powers and emerging economies are actively contributing to the clinical development of novel cartilage repair technologies, reinforcing the progressive internationalization of translational research in joint regeneration. These comparative findings are summarized in Figure 9a–c, which illustrates the global distribution of scientific publications, clinical studies, and patent families across countries and territories.

The integration of bibliometric, technological, and clinical evidence indicates that articular cartilage repair has evolved into a highly multidisciplinary and translational field in which advances in basic science, biomaterials engineering, and orthopedic surgery converge. While scientific publications and preclinical studies demonstrate a robust and mature knowledge base, particularly in scaffolds, biomaterials, and stem cell biology, the pace of clinical translation and regulatory approval remains comparatively slow. This imbalance reflects the intrinsic complexity of cartilage regeneration, as promising laboratory outcomes often face challenges related to reproducibility, safety, cost-effectiveness, and long-term functional performance in clinical settings. Importantly, the volume of scientific production should not be interpreted as a surrogate for clinical maturity, as only a limited number of these technologies have demonstrated reproducible long-term benefit in patients.

The observed imbalance between rapid scientific and technological innovation and comparatively slow clinical adoption also reflects the long maturation timelines inherent to cartilage repair. Unlike other musculoskeletal interventions, meaningful evaluation of cartilage regeneration requires extended follow-up to assess tissue stability, integration, and resistance to mechanical loading over time. Consequently, promising preclinical and early clinical results may take years to translate into standard clinical practice, contributing to the apparent disconnect between publication growth, patent activity, and the availability of clinically established therapies.

Undoubtedly, ATMPs have emerged as a promising alternative in cartilage restoration, as results in preclinical models demonstrate. However, challenges such as regulatory requirements, clinical translation, and operating costs could delay short-term access to these therapies. In addition, variability across clinical trials further complicates direct comparison between competing technologies. In fact, outcomes following MSC-based therapies differ substantially depending on cell source (bone marrow, adipose tissue, synovium), expansion protocols, delivery method, and lesion characteristics, resulting in heterogeneous structural and functional results across studies [129]. Other therapies, such as PRP, although widely used in clinical settings, require a robust standardization to enable reproducibility of manufacturing conditions and reduce variability in its composition. It has been reported that PRP interventions show considerable inter-study variability due to differences in leukocyte content, activation methods, and dosing schedules, and limiting reproducibility [13]. In this context, allografts and osteochondral systems are the alternatives with the greatest therapeutic advantages, owing to their high biocompatibility, structural support for tissue formation, and widespread use in surgical practice. Nevertheless, factors such as dependence on donations could significantly limit the adoption of this technology.

In this context, recent advances in advanced biomaterials and biofabrication platforms, including microfluidics, electrospinning, and bioprinting, have enabled unprecedented control over scaffold architecture, spatial cell distribution, and biochemical functionalization. Major achievements include the repair of hyaline-like cartilage and long-term functional integration using biomimetic scaffolds based on collagen, hyaluronic acid, or hybrid systems in large animal models, as well as mechanically robust and highly tunable PCL- and PLGA-based platforms that promote chondrogenesis and osteochondral repair in vivo. Emerging nanocomposite and piezoelectric scaffolds further represent a significant advance by enabling mechanical–electrical stimulation of cells and enhancing extracellular matrix deposition without the need for external devices. However, clinical translation remains constrained by the lack of standardized head-to-head comparisons between materials, inconsistent long-term in vivo validation under physiological joint loading, and limited reporting of failure modes such as poor integration or fibrocartilaginous repair. In addition, the lack of standardized fabrication protocols and inter-laboratory validation studies further limits the reproducibility of these technologies and complicates their comparison across preclinical and clinical settings. Furthermore, although in situ and cell-based bioprinting strategies show superior biological performance and patient-specific repair potential, their manufacturing complexity, scalability limitations, regulatory burden, and cost are rarely addressed at the preclinical stage, constituting a critical barrier to clinical adoption.

Within the landscape of emerging scaffold-based therapies, electroactive biomaterials—particularly piezoelectric hydrogels—have introduced a new paradigm in cartilage regeneration by enabling the conversion of physiological mechanical loading into endogenous electrical stimulation. Most studies to date have focused on nanoparticle-reinforced nanocomposite systems incorporating piezoelectric phases such as BaTiO_3_, ZnO, or PVDF nanostructures, which have been shown to enhance mesenchymal stem cell chondrogenesis, increase glycosaminoglycan and type II collagen deposition, and modulate inflammatory signaling [130,131,132]. These findings support the concept that bioelectromechanical cues play a central role in recapitulating the functional microenvironment of native cartilage.

More recently, intrinsically piezoelectric hydrogels based on electroactive polymer networks without inorganic nanofillers have emerged as a distinct and potentially more translationally feasible strategy [133,134]. In vivo studies have shown that these systems can recruit endogenous progenitor cells, activate TGF-β–mediated pathways, and promote the formation of hyaline-like cartilage in osteochondral defect models while simultaneously modulating the osteoarthritic inflammatory microenvironment [69]. Their injectability, structural homogeneity, and capacity for non-invasive activation (e.g., ultrasound-mediated stimulation) position them as attractive candidates for cell-free and minimally invasive therapeutic approaches. From a translational standpoint, the absence of dispersed inorganic nanoparticles may reduce long-term biosafety concerns, improve manufacturing reproducibility, and simplify regulatory classification compared with nanocomposite counterparts.

Despite these promising biological and functional outcomes, electroactive hydrogel systems remain at an early stage of clinical translation. Current evidence is largely limited to short-term in vitro experiments and small-animal models, and direct benchmarking against clinically established strategies such as ACI/MACI, osteochondral grafting, or advanced non-electroactive scaffolds remains lacking. Consequently, their future clinical relevance will depend not only on their bioelectrical functionality but also on demonstrating long-term mechanical stability, scalable, GMP-compatible fabrication, and reproducible therapeutic performance in large-animal and comparative studies.

Despite these technological advances, a persistent misalignment exists between the sophistication of emerging biomaterials and the clinical endpoints commonly used to evaluate their performance. Most clinical studies continue to rely on pain scores and functional outcome measures, which may not adequately capture improvements in tissue quality, biomechanical restoration, or long-term durability. As a result, the added value of complex scaffold architectures, biofunctionalization strategies, or smart materials may remain underrepresented in clinical trials, limiting their perceived benefit and slowing regulatory approval and reimbursement decisions.

A critical challenge in cartilage repair lies in the marked heterogeneity of the available clinical evidence. Head-to-head comparative clinical studies remain scarce; however, when outcomes are analyzed across systematic reviews and long-term follow-up cohorts, cell-based and osteochondral grafting techniques consistently demonstrate superior structural repair and durability compared with bone marrow stimulation approaches. Although microfracture remains widely adopted because of its simplicity, low cost, and minimal invasiveness, long-term follow-up studies consistently report the formation of fibrocartilaginous repair tissue with inferior biomechanical properties and progressive clinical deterioration when compared with cell-based or scaffold-assisted techniques. In contrast, advanced strategies such as ACI/MACI and osteochondral grafting exhibit superior regenerative potential and more durable clinical outcomes, but their widespread adoption is limited by higher costs, technical complexity, and stringent regulatory requirements. On the other hand, advanced biofabrication platforms, including multifunctional scaffolds, smart biomaterials, and in situ bioprinting systems, demonstrate substantial preclinical promise but remain at an early or intermediate translational stage, with unresolved challenges related to scalability, regulatory classification, and cost-effectiveness. This differentiation underscores the gap between technological sophistication and clinical consolidation within the field.

From a translational perspective, patent activity highlights intense innovation and competition in the field; nevertheless, the high proportion of inactive or non-commercialized patents indicates persistent barriers to translating technological advances into clinically viable products, largely driven by regulatory hurdles and health-system cost–benefit constraints. Notably, cell-free approaches combined with advanced biomaterials are gaining increasing traction in cartilage repair; however, further refinement of regulatory pathways and translational frameworks will be necessary to enable large-scale clinical access. Moreover, several biomaterial-based platforms that demonstrated strong preclinical chondrogenic potential have failed to progress beyond early-phase clinical trials or have been discontinued because of manufacturing complexity, regulatory burden, or insufficient long-term benefit, and in some cases have shown loss of efficacy or inconsistent outcomes in early clinical evaluation, leading to discontinuation of further development. This highlights that biological performance in controlled experimental environments does not necessarily predict clinical success.

Beyond heterogeneity in evidence quality, an additional challenge lies in the absence of universally accepted criteria for matching specific cartilage repair technologies to well-defined clinical scenarios. Defect size, depth, anatomical location, and containment, together with patient-related factors such as age, activity level, and joint environment, critically influence treatment success. While bone marrow stimulation techniques are commonly selected for small focal defects, larger or osteochondral lesions often require scaffold-based, cell-assisted, or grafting strategies. However, comparative clinical data supporting clear decision thresholds remain limited, contributing to variability in clinical practice and reinforcing the need for stratified, indication-driven treatment algorithms. This comparative, maturity-oriented interpretation allows the different technologies to be evaluated not as parallel alternatives but as interventions at distinct stages of clinical consolidation, with different levels of evidence, reproducibility, and long-term success.

This review also has inherent limitations. The analysis is dependent on the scope and indexing of the selected scientific, patent, and clinical databases, which may underrepresent unpublished data or region-specific innovations. In addition, the rapid evolution of regenerative technologies means that some emerging approaches may still lack sufficient clinical evidence for comprehensive comparison. Finally, differences in regulatory frameworks and clinical practice patterns across regions may limit the generalizability of certain conclusions.

Collectively, the evidence supports a shift toward integrative and multimodal therapeutic strategies in which biological therapies (e.g., MSCs, PRP, and exosomes), advanced biomaterial scaffolds, and optimized surgical approaches are not viewed as competing alternatives but as complementary components of a unified regenerative paradigm. Achieving this transition will require sustained collaboration among academia, industry, clinicians, and regulatory agencies to effectively bridge the gap between technological innovation and patient access.

Nevertheless, despite the unprecedented technological sophistication of cartilage repair research, translational success has progressed at a considerably slower pace. The discrepancy between experimental innovation and durable clinical outcomes reflects a structural challenge within the field: biological plausibility does not necessarily translate into therapeutic scalability. Highly engineered constructs frequently demonstrate remarkable preclinical performance, yet encounter critical barriers related to GMP-compliant manufacturing, regulatory classification, reproducibility, cost, and long-term safety validation. Consequently, the future of cartilage repair will not be defined solely by material complexity or biofabrication precision but by the convergence of mechanistic robustness, clinical reproducibility, and translational pragmatism. Establishing hierarchies of maturity and feasibility is therefore essential to prevent technological enthusiasm from outpacing clinical reality.

## 5. Conclusions

The management of articular cartilage injuries has progressively evolved from conventional surgical techniques, such as microfracture and osteochondral grafting, toward integrative strategies that combine advanced biomaterials, tissue engineering, and cell-based therapies. In this contemporary paradigm, surgery is no longer limited to defect repair alone but is increasingly adapted to enable the effective implementation of regenerative technologies. These approaches may include arthroscopic procedures, open or mini-open surgery, intra-articular injections, or a combination of techniques. The selection of the most appropriate intervention is guided by patient-specific factors, lesion characteristics, and the clinical and regulatory maturity of each technology, reflecting a landscape in which established and emerging procedures converge to enhance functional cartilage restoration.

Articular cartilage repair thus represents one of the most dynamic, yet challenging, areas within regenerative medicine. This review demonstrates that while scientific output has grown steadily and patent activity is substantial, clinical validation and widespread adoption remain heterogeneous. Comparative analyses indicate that the field is currently in a phase of consolidation and selective translation, where certain technologies, such as MACI, osteochondral grafts, OCA systems, and biofunctionalized scaffolds, have achieved clinical implementation, whereas others remain confined to experimental or early translational stages.

Within this context, OCA techniques emerge as a particularly relevant option for the treatment of large chondral and osteochondral defects, offering the unique advantage of restoring both hyaline cartilage and subchondral bone in a single surgical procedure. Although their broader application is limited by donor availability, graft-preservation constraints, and integration challenges, OCAs provide valuable therapeutic alternatives in complex cases where bone marrow-stimulation techniques (microfracture) or cell-based therapies alone may be insufficient. Their increasing incorporation into clinical practice highlights the importance of integrating established surgical solutions with emerging regenerative strategies.

In parallel, tissue engineering approaches and biomaterial-based platforms, including scaffolds, hydrogels, and three-dimensional bioprinter matrices, are consolidating as a central axis of translational research. These systems not only support cellular therapies but also enable the development of patient-specific implants with tailored degradation profiles, bioactivity, and mechanical properties that more closely approximate those of native cartilage. When combined with stem cells or bioactive molecules, these platforms represent one of the most promising pathways toward durable, functionally effective cartilage regeneration.

Future progress in the field will depend on addressing several critical challenges:

- Standardization of manufacturing and clinical protocols for cell-based therapies and biomaterials.
- Long-term clinical follow-up to evaluate tissue durability, functional outcomes, and integration.
- Comprehensive cost-effectiveness analyses to facilitate broader adoption within healthcare systems.
- The development of personalized and multimodal treatment strategies that integrate advanced biomaterials, tissue engineering platforms, OCA systems, and biological therapies tailored to individual patient needs.

In conclusion, articular cartilage repair is transitioning from experimental promise toward clinical reality. Its consolidation as a standard of care will require coordinated advances in basic science, biomaterials and tissue engineering, surgical innovation, including osteochondral grafting strategies, industrial scale-up, and regulatory harmonization. Only through this integrated, translational effort can scientific progress be translated into safe, effective, and accessible therapies that ultimately improve patient outcomes and quality of life.

## Figures and Tables

**Table 1:** Translational Evaluation Matrix for Technological Advances in Articular Cartilage Regenerative Medicine.

Technology/Technique	Surgical Technique/Application Mode	Main Application	Advantages	Limitations/Challenges	Evidence Level/Development Stage	Relevant Actors for the Review
Scaffolds (polymers, 3D printing, composites)	Implantation via arthroscopy or open surgery	Structural support for cartilage regeneration	Biocompatible, supports cells, customizable via 3D printing	Uncontrolled degradation, incomplete integration	Preclinical/Early clinical	Tepha Inc. (Lexington, MA, USA), MIT (Cambridge, MA, USA), Univ. of Leeds (Leeds, UK) [108,109]
Hydrogels (injectable, bioactive)	Direct intra-articular injection or arthroscopic delivery	Matrices for cell/drug encapsulation	High water retention, adaptable to soft tissues	Low mechanical strength, stability issues	Preclinical/Early trials	Brigham & Women’s Hospital (Boston, MA, USA), Selecta Biosciences (Watertown, MA, USA) [110,111]
ACI	Two-stage open or arthroscopic procedure	Restoration with patient’s own cells	Avoids immune rejection, good long-term function	High cost, risk of delamination, complex rehab	High (comparative studies, RCTs)	Vericel Corp. (Cambridge, MA, USA), FDA (Silver Spring, MD, USA) approval [112,113]
MACI	Scaffold-based chondrocyte implantation with sutures/adhesives	Functional regeneration of hyaline cartilage	High patient satisfaction, better defect filling	Expensive, requires intensive rehab	Moderate–High (controlled prospective studies)	Vericel Corp., FDA [114,115]
Microfracture/Microperforations	Arthroscopic bone marrow stimulation	Induction of MSCs migration for cartilage repair	Minimally invasive, inexpensive, reproducible	Fibrocartilage of lower quality, short-term effect	Variable (Level I–IV studies)	Broad clinical use, gold standard baseline [116]
OATS/Mosaicplasty (osteochondral autograft)	Open or mini-open surgery	Focal defect repair with autologous grafts	Integration with hyaline cartilage, good outcomes	Donor site morbidity, limited graft availability	Moderate evidence	Mayo Clinic (Rochester, MN, USA), tissue banks [117]
Osteochondral Allograft	Open implantation of donor grafts	Repair of large osteochondral defects	No donor morbidity, large coverage	Risk of resorption, immune rejection, limited availability	Moderate	Cleveland Clinic (Cleveland, OH, USA), Hospital for Special Surgery (New York, NY, USA) [117]
OCA (Osteochondral Cylinder/Allograft/Autograft Systems)	Open surgery with modular grafts, sometimes combined with scaffolds	Extensive cartilage defect repair; joint preservation	Immediate biomechanical function, hyaline cartilage + subchondral bone replacement	Limited donor availability, storage issues, variable integration	Moderate, growing use in clinical practice	Cleveland Clinic, HSS, tissue banks, biotech collaborations [118]
Biological Augmentation (MSCs, PRP, Exosomes, Gene Therapy)	Injection (guided), arthroscopy, or combined with scaffold	Cell-based or cell-free regenerative therapy	High regenerative potential, immunomodulatory effects	Still experimental, regulatory and safety challenges	Early clinical trials/Preclinical	MIT, Virginia Commonwealth Univ. (Richmond, VA, USA), multiple biotech companies [119]

## Data Availability

No new data were created or analyzed in this study. Data sharing is not applicable to this article.

## Abbreviations

The following abbreviations are used in this manuscript:

OA	Osteoarthritis
NSAIDs	Nonsteroidal Anti-Inflammatory Drugs
3D	Three-Dimensional
ACI	Autologous Chondrocyte Implantation
CPC	Cooperative Patent Classification
IPC	International Patent Classification
EPO	European Patent Office
WIPO	World Intellectual Property Organization
MCP	Macromolecular Chemistry Polymers
PRP	Platelet-Rich Plasma
BMAC	Bone Marrow Aspirate Concentrate
MCM	Micronized Allogeneic Cartilage Matrix
MSCs	Mesenchymal Stromal Cells
TGF-β	Transforming Growth Factor Beta
BMP-2	Bone Morphogenetic Protein-2
IGF-1	Insulin-Like Growth Factor-1
EVs	Extracellular Vesicles
miRNAs	Micrornas
lncRNAs	Long Non-Coding Rnas
circRNAs	Circular Rnas
SEC	Size Exclusion Chromatography
RA	Rheumatoid Arthritis
MPs	Microparticles
DTH	Delayed-Type Hypersensitivity
CIA	Collagen-Induced Arthritis
ECM	Extracellular Matrix
hUC-MSCs	Human Umbilical Cord–Derived Mscs
PLGA	Poly(Lactide-Co-Glycolide)
dECM	Decellularized Extracellular Matrix
DCM	Decellularized Cartilage Matrix
OATS	Osteochondral Autograft Transfer Systems
OCAs	Osteochondral Allografts
HA	Hyaluronic acid
PCL	Poly (ε-caprolactone)
tFNAs	Tetrahedral framework nucleic acids
PLA	Polylactic acid
FDM	Fused deposition modeling
CAD	Computer-aided design
MRI	Magnetic resonance imaging
CT	Computed tomography
PLCL	Poly(L-lactide-co-ε-caprolactone)
GAGs	Glycosaminoglycans
AAOS	American Academy of Orthopaedic Surgeons
MACI	Matrix-induced autologous chondrocyte implantation
AMIC	Autologous matrix-induced chondrogenesis
ATMPs	Advanced therapy medicinal products
FDA	Food and Drug Administration
EMA	European Medicines Agency
GMP	Good Manufacturing Practices
IL-4	Interleukin 4
BaTiO_3_	Barium titanate
ZnO	Zinc oxide
PVDF	Poly(vinylidene fluoride)

## References

[1] Zhang R., Chang S.J., Jing Y., Wang L., Chen C.-J., Liu J.-T. (2023). Application of Chitosan with Different Molecular Weights in Cartilage Tissue Engineering. Carbohydr. Polym..

[2] ur Rehman S., Iqbal S., Shahid M.U., Jahangir M.S., Malik A.L. (2024). Cartilage: Structure, Function, and the Pathogenesis of Osteoarthritis. Advancements in Synovial Joint Science—Structure, Function, and Beyond.

[3] Kheir E., Shaw D. (2009). Hyaline Articular Cartilage. Orthop. Trauma.

[4] Sophia Fox A.J., Bedi A., Rodeo S.A. (2009). The Basic Science of Articular Cartilage: Structure, Composition, and Function. Sports Health.

[5] Yang C., Chen R., Chen C., Yang F., Xiao H., Geng B., Xia Y. (2024). Tissue Engineering Strategies Hold Promise for the Repair of Articular Cartilage Injury. Biomed. Eng. Online.

[6] Roseti L., Desando G., Cavallo C., Petretta M., Grigolo B. (2019). Articular Cartilage Regeneration in Osteoarthritis. Cells.

[7] Pourakbari R., Khodadadi M., Aghebati-Maleki A., Aghebati-Maleki L., Yousefi M. (2019). The Potential of Exosomes in the Therapy of the Cartilage and Bone Complications; Emphasis on Osteoarthritis. Life Sci..

[8] Focsa M.A., Florescu S., Gogulescu A. (2025). Emerging Strategies in Cartilage Repair and Joint Preservation. Medicina.

[9] Chen M., Jiang Z., Zou X., You X., Cai Z., Huang J. (2024). Advancements in Tissue Engineering for Articular Cartilage Regeneration. Heliyon.

[10] Pina S., Ribeiro V.P., Marques C.F., Maia F.R., Silva T.H., Reis R.L., Oliveira J.M. (2019). Scaffolding Strategies for Tissue Engineering and Regenerative Medicine Applications. Mater. Basel Switz..

[11] Andriolo L., Reale D., Di Martino A., Boffa A., Zaffagnini S., Filardo G. (2021). Cell-Free Scaffolds in Cartilage Knee Surgery: A Systematic Review and Meta-Analysis of Clinical Evidence. Cartilage.

[12] Jin P., Liu H., Chen X., Liu W., Jiang T. (2024). From Bench to Bedside: The Role of Extracellular Vesicles in Cartilage Injury Treatment. Biomater. Res..

[13] Mithoefer K., McAdams T., Williams R.J., Kreuz P.C., Mandelbaum B.R. (2009). Clinical Efficacy of the Microfracture Technique for Articular Cartilage Repair in the Knee: An Evidence-Based Systematic Analysis. Am. J. Sports Med..

[14] Negoro T., Takagaki Y., Okura H., Matsuyama A. (2018). Trends in Clinical Trials for Articular Cartilage Repair by Cell Therapy. npj Regen. Med..

[15] Chahal J., Gross A.E., Gross C., Mall N., Dwyer T., Chahal A., Whelan D.B., Cole B.J. (2013). Outcomes of Osteochondral Allograft Transplantation in the Knee. Arthrosc. J. Arthrosc. Relat. Surg..

[16] Lara-Bertrand A.L., Camelo F., Camacho B., Silva-Cote I. (2024). Technological Search of Patents for the Identification of Devices with Potential Use in Tumor-Infiltrating Lymphocytes (TILs) Research. World Pat. Inf..

[17] Singer J., Knezic N., Layne J., Gohring G., Christiansen J., Rothrauff B., Huard J. (2024). Enhancing Cartilage Repair: Surgical Approaches, Orthobiologics, and the Promise of Exosomes. Life.

[18] Cong B., Sun T., Zhao Y., Chen M. (2023). Current and Novel Therapeutics for Articular Cartilage Repair and Regeneration. Ther. Clin. Risk Manag..

[19] Frederico C., Conceição A., Nóbrega C., Mendonça L.S. (2025). Advanced Therapy Medicinal Products Development—From Guidelines to Medicines in the Market. Biotechnol. Adv..

[20] Pharoun J., Berro J., Sobh J., Abou-Younes M.-M., Nasr L., Majed A., Khalil A., Stephan J., Faour W.H. (2024). Mesenchymal Stem Cells Biological and Biotechnological Advances: Implications for Clinical Applications. Eur. J. Pharmacol..

[21] Pînzariu A.C., Moscalu R., Soroceanu R.P., Maranduca M.A., Drochioi I.C., Vlasceanu V.I., Timofeiov S., Timofte D.V., Huzum B., Moscalu M. (2025). The Therapeutic Use and Potential of MSCs: Advances in Regenerative Medicine. Int. J. Mol. Sci..

[22] Prodromos C., Rumschlag T. (2021). Administration of Autologous Mesenchymal Cells for the Treatment of Arthritis. Curr. Stem Cell Res. Ther..

[23] de Windt T.S., Vonk L.A., Slaper-Cortenbach I.C., van den Broek M.P., Nizak R., van Rijen M.H., de Weger R.A., Dhert W.J., Saris D.B. (2017). Allogeneic Mesenchymal Stem Cells Stimulate Cartilage Regeneration and Are Safe for Single-Stage Cartilage Repair in Humans upon Mixture with Recycled Autologous Chondrons. Stem Cells.

[24] Mastbergen S.C., Saris D.B., Lafeber F.P. (2013). Functional Articular Cartilage Repair: Here, near, or Is the Best Approach Not yet Clear?. Nat. Rev. Rheumatol..

[25] Musiał-Wysocka A., Kot M., Majka M. (2019). The Pros and Cons of Mesenchymal Stem Cell-Based Therapies. Cell Transplant..

[26] Brittberg M., Lindahl A., Nilsson A., Ohlsson C., Isaksson O., Peterson L. (1994). Treatment of Deep Cartilage Defects in the Knee with Autologous Chondrocyte Transplantation. N. Engl. J. Med..

[27] Huang H., Xu H., Zhang J., Huang H., Xu H., Zhang J. (2019). Current Tissue Engineering Approaches for Cartilage Regeneration. Cartilage Tissue Engineering and Regeneration Techniques.

[28] Vinatier C., Guicheux J. (2016). Cartilage Tissue Engineering: From Biomaterials and Stem Cells to Osteoarthritis Treatments. Ann. Phys. Rehabil. Med..

[29] Kamusheva M., Turcu-Stiolica A., Gierczyński J., Subtirelu M.-S., Czech M., Petrova G. (2021). Do Advanced Therapies Have a Future in the Low- and Middle-Income Countries—The Case of Bulgaria, Romania, and Poland. Front. Public Health.

[30] Liu Y., Ma Y., Zhang J., Yuan Y., Wang J. (2019). Exosomes: A Novel Therapeutic Agent for Cartilage and Bone Tissue Regeneration. Dose-Response.

[31] Longfei H., Wenyuan H., Weihua F., Peng P., Sun L., Kun L., Mincong H., Fan Y., Wei H., Qiushi W. (2025). Exosomes in Cartilage Microenvironment Regulation and Cartilage Repair. Front. Cell Dev. Biol..

[32] Zhou Q., Cai Y., Jiang Y., Lin X. (2020). Exosomes in Osteoarthritis and Cartilage Injury: Advanced Development and Potential Therapeutic Strategies. Int. J. Biol. Sci..

[33] Cosenza S., Toupet K., Maumus M., Luz-Crawford P., Blanc-Brude O., Jorgensen C., Noël D. (2018). Mesenchymal Stem Cells-Derived Exosomes Are More Immunosuppressive than Microparticles in Inflammatory Arthritis. Theranostics.

[34] Zhang S., Wong K.L., Ren X., Teo K.Y.W., Afizah H., Choo A.B.H., Lai R.C., Lim S.K., Hui J.H.P., Toh W.S. (2022). Mesenchymal Stem Cell Exosomes Promote Functional Osteochondral Repair in a Clinically Relevant Porcine Model. Am. J. Sports Med..

[35] Yang D., Yang J., Chang S.-J., Hu J.-L., Chen Y.-J., Yang S.-W. (2025). Exosome-Seeded Cryogel Scaffolds for Extracellular Matrix Regeneration in the Repair of Articular Cartilage Defects: An In Vitro and In Vivo Rabbit Model Study. Polymers.

[36] Liang Y., Li J., Wang Y., He J., Chen L., Chu J., Wu H. (2022). Platelet Rich Plasma in the Repair of Articular Cartilage Injury: A Narrative Review. Cartilage.

[37] Tian G., Jiang S., Li J., Wei F., Li X., Ding Y., Yang Z., Sun Z., Zha K., Wang F. (2021). Cell-Free Decellularized Cartilage Extracellular Matrix Scaffolds Combined with Interleukin 4 Promote Osteochondral Repair through Immunomodulatory Macrophages: In Vitro and in Vivo Preclinical Study. Acta Biomater..

[38] Marmotti A., Rossi R., Castoldi F., Roveda E., Michielon G., Peretti G.M. (2015). PRP and Articular Cartilage: A Clinical Update. BioMed Res. Int..

[39] Xie X., Zhang C., Tuan R.S. (2014). Biology of Platelet-Rich Plasma and Its Clinical Application in Cartilage Repair. Arthritis Res. Ther..

[40] Slimi F., Zribi W., Trigui M., Amri R., Gouiaa N., Abid C., Rebai M.A., Boudawara T., Jebahi S., Keskes H. (2021). The Effectiveness of Platelet-Rich Plasma Gel on Full-Thickness Cartilage Defect Repair in a Rabbit Model. Bone Jt. Res..

[41] Zhang Y., Niu J., Wang Z., Liu S., Wu J., Yu B. (2017). Repair of Osteochondral Defects in a Rabbit Model Using Bilayer Poly (Lactide-Co-Glycolide) Scaffolds Loaded with Autologous Platelet-Rich Plasma. Med. Sci. Monit..

[42] Mifune Y., Matsumoto T., Takayama K., Ota S., Li H., Meszaros L.B., Usas A., Nagamune K., Gharaibeh B., Fu F.H. (2013). The Effect of Platelet-Rich Plasma on the Regenerative Therapy of Muscle Derived Stem Cells for Articular Cartilage Repair. Osteoarthr. Cartil..

[43] Abedin E., Lari R., Mahdavi Shahri N., Fereidoni M. (2018). Development of a Demineralized and Decellularized Human Epiphyseal Bone Scaffold for Tissue Engineering: A Histological Study. Tissue Cell.

[44] Zhang Y., Jiang L., Zheng T., Sha L., Wang J., Dong H., Song K., Liu T. (2019). Development of Decellularized Meniscus Extracellular Matrix and Gelatin/Chitosan Scaffolds for Meniscus Tissue Engineering. Biomed. Mater. Eng..

[45] Ghazanfari S., Alberti K.A., Xu Q., Khademhosseini A. (2019). Evaluation of an Elastic Decellularized Tendon-Derived Scaffold for the Vascular Tissue Engineering Application. J. Biomed. Mater. Res. A.

[46] Parmaksiz M., Elçin A.E., Elçin Y.M. (2019). Decellularized Bovine Small Intestinal Submucosa-PCL/Hydroxyapatite-Based Multilayer Composite Scaffold for Hard Tissue Repair. Mater. Sci. Eng. C Mater. Biol. Appl..

[47] Grant R., Hallett J., Forbes S., Hay D., Callanan A. (2019). Blended Electrospinning with Human Liver Extracellular Matrix for Engineering New Hepatic Microenvironments. Sci. Rep..

[48] Barthold J.E., Cai L., McCreery K.P., Fischenich K.M., Eckstein K.N., Ferguson V.L., Emery N.C., Breur G., Neu C.P. (2024). Integrative Cartilage Repair Using Acellular Allografts for Engineered Structure and Surface Lubrication in Vivo. npj Regen. Med..

[49] Lee B.-S., Kim H.-J., Lee C.-R., Bin S.-I., Lee D.-H., Kim N.-J., Kim C.-W. (2018). Clinical Outcomes of Meniscal Allograft Transplantation with or Without Other Procedures: A Systematic Review and Meta-Analysis. Am. J. Sports Med..

[50] Brittberg M. (2010). Cell Carriers as the Next Generation of Cell Therapy for Cartilage Repair: A Review of the Matrix-Induced Autologous Chondrocyte Implantation Procedure. Am. J. Sports Med..

[51] Lima E.G., Grace Chao P.-H., Ateshian G.A., Bal B.S., Cook J.L., Vunjak-Novakovic G., Hung C.T. (2008). The Effect of Devitalized Trabecular Bone on the Formation of Osteochondral Tissue-Engineered Constructs. Biomaterials.

[52] Rowland R., Colello M., Wyland D.J. (2019). Osteochondral Autograft Transfer Procedure: Arthroscopic Technique and Technical Pearls. Arthrosc. Technol..

[53] Toh W.S., Spector M., Lee E.H., Cao T. (2011). Biomaterial-Mediated Delivery of Microenvironmental Cues for Repair and Regeneration of Articular Cartilage. Mol. Pharm..

[54] Moroni L., Boland T., Burdick J.A., Maria C.D., Derby B., Forgacs G., Groll J., Li Q., Malda J., Mironov V.A. (2018). Biofabrication: A Guide to Technology and Terminology. Trends Biotechnol..

[55] Frassica M.T., Grunlan M.A. (2020). Perspectives on Synthetic Materials to Guide Tissue Regeneration for Osteochondral Defect Repair. ACS Biomater. Sci. Eng..

[56] Iulian A., Dan L., Camelia T., Claudia M., Sebastian G., Oliveira J.M., Pina S., Reis R.L., San Roman J. (2018). Synthetic Materials for Osteochondral Tissue Engineering. Osteochondral Tissue Engineering.

[57] Cheng L., Tong X., Li Z., Liu Z., Huang H., Zhao H., Dai F. (2018). Natural Silkworm Cocoon Composites with High Strength and Stiffness Constructed in Confined Cocooning Space. Polymers.

[58] Foroughi A.H., Razavi M.J. (2022). Multi-Objective Shape Optimization of Bone Scaffolds: Enhancement of Mechanical Properties and Permeability. Acta Biomater..

[59] Ciritsis A., Horbach A., Staat M., Kuhl C.K., Kraemer N.A. (2018). Porosity and Tissue Integration of Elastic Mesh Implants Evaluated in Vitro and in Vivo. J. Biomed. Mater. Res. B Appl. Biomater..

[60] Skotak M., Noriega S., Larsen G., Subramanian A. (2010). Electrospun Cross-Linked Gelatin Fibers with Controlled Diameter: The Effect of Matrix Stiffness on Proliferative and Biosynthetic Activity of Chondrocytes Cultured in Vitro. J. Biomed. Mater. Res. A.

[61] Fan S., Chen K., Yuan W., Zhang D., Yang S., Lan P., Song L., Shao H., Zhang Y. (2020). Biomaterial-Based Scaffolds as Antibacterial Suture Materials. ACS Biomater. Sci. Eng..

[62] Theodoridis K., Manthou M.E., Aggelidou E., Kritis A. (2022). In Vivo Cartilage Regeneration with Cell-Seeded Natural Biomaterial Scaffold Implants: 15-Year Study. Tissue Eng. Part B Rev..

[63] Sennett M.L., Friedman J.M., Ashley B.S., Stoeckl B.D., Patel J.M., Alini M., Cucchiarini M., Eglin D., Madry H., Mata A. (2021). Long Term Outcomes of Biomaterial-Mediated Repair of Focal Cartilage Defects in A Large Animal Model. Eur. Cells Mater..

[64] Liu Q., Dai W., Gao Y., Li S., Zhao X., Jia H., Tan Y., Guo L., Fan Y., Zhang X. (2025). The Degradation Rate of Collagen-Based Hydrogels Regulates Chondrogenic Differentiation of Bone Marrow Mesenchymal Stem Cells. Collagen Leather.

[65] Lv J., Wu Y., Cao Z., Liu X., Sun Y., Zhang P., Zhang X., Tang K., Cheng M., Yao Q. (2023). Enhanced Cartilage and Subchondral Bone Repair Using Carbon Nanotube-Doped Peptide Hydrogel–Polycaprolactone Composite Scaffolds. Pharmaceutics.

[66] Najjari A., Aghdam R.M., Ebrahimi S.A.S., Suresh K.S., Krishnan S., Shanthi C., Ramalingam M. (2022). Smart Piezoelectric Biomaterials for Tissue Engineering and Regenerative Medicine: A Review. Biomed. Eng. Biomed. Technol..

[67] Wang X., Stefanello S.T., Shahin V., Qian Y. (2025). From Mechanoelectric Conversion to Tissue Regeneration: Translational Progress in Piezoelectric Materials. Adv. Mater..

[68] Ni X., Xing X., Deng Y., Li Z. (2023). Applications of Stimuli-Responsive Hydrogels in Bone and Cartilage Regeneration. Pharmaceutics.

[69] Vinikoor T., Dzidotor G.K., Le T.T., Liu Y., Kan H.-M., Barui S., Chorsi M.T., Curry E.J., Reinhardt E., Wang H. (2023). Injectable and Biodegradable Piezoelectric Hydrogel for Osteoarthritis Treatment. Nat. Commun..

[70] Kalkan R., Nwekwo C.W., Adali T. (2018). The Use of Scaffolds in Cartilage Regeneration. Crit. Rev. Eukaryot. Gene Expr..

[71] Li P., Fu L., Liao Z., Peng Y., Ning C., Gao C., Zhang D., Sui X., Lin Y., Liu S. (2021). Chitosan Hydrogel/3D-Printed Poly(Ε-caprolactone) Hybrid Scaffold Containing Synovial Mesenchymal Stem Cells for Cartilage Regeneration Based on Tetrahedral Framework Nucleic Acid Recruitment. Biomaterials.

[72] Zhang Y., Yang F., Liu K., Shen H., Zhu Y., Zhang W., Liu W., Wang S., Cao Y., Zhou G. (2012). The Impact of PLGA Scaffold Orientation on in Vitro Cartilage Regeneration. Biomaterials.

[73] Rezuş E., Burlui A., Cardoneanu A., Macovei L.A., Tamba B.I., Rezuş C. (2021). From Pathogenesis to Therapy in Knee Osteoarthritis: Bench-to-Bedside. Int. J. Mol. Sci..

[74] Makris E.A., Gomoll A.H., Malizos K.N., Hu J.C., Athanasiou K.A. (2015). Repair and Tissue Engineering Techniques for Articular Cartilage. Nat. Rev. Rheumatol..

[75] Haghighi P., Shamloo A. (2021). Fabrication of a Novel 3D Scaffold for Cartilage Tissue Repair: In-Vitro and in-Vivo Study. Mater. Sci. Eng. C.

[76] Deng L., Liu Y., Wu Q., Lai S., Yang Q., Mu Y., Dong M. (2024). Exosomes to Exosome-Functionalized Scaffolds: A Novel Approach to Stimulate Bone Regeneration. Stem Cell Res. Ther..

[77] Teo B.K.K., Wong S.T., Lim C.K., Kung T.Y.S., Yap C.H., Ramagopal Y., Romer L.H., Yim E.K.F. (2013). Nanotopography Modulates Mechanotransduction of Stem Cells and Induces Differentiation through Focal Adhesion Kinase. ACS Nano.

[78] Naderi-Meshkin H., Andreas K., Matin M.M., Sittinger M., Bidkhori H.R., Ahmadiankia N., Bahrami A.R., Ringe J. (2014). Chitosan-based Injectable Hydrogel as a Promising in Situ Forming Scaffold for Cartilage Tissue Engineering. Cell Biol. Int..

[79] Wang Y., Pereira R.F., Peach C., Huang B., Vyas C., Bartolo P. (2023). Robotic in Situ Bioprinting for Cartilage Tissue Engineering. Int. J. Extrem. Manuf..

[80] Owida H.A., Mohammad S.I., Al Oraini B., Vasudevan A. (2025). Advances in Handheld 4D Bioprinting for In Situ Cartilage Tissue Engineering: Materials, Techniques, and Clinical Potential. Regen. Eng. Transl. Med..

[81] Di Bella C., Duchi S., O’Connell C.D., Blanchard R., Augustine C., Yue Z., Thompson F., Richards C., Beirne S., Onofrillo C. (2018). In Situ Handheld Three-Dimensional Bioprinting for Cartilage Regeneration. J. Tissue Eng. Regen. Med..

[82] Roseti L., Cavallo C., Desando G., Parisi V., Petretta M., Bartolotti I., Grigolo B. (2018). Three-Dimensional Bioprinting of Cartilage by the Use of Stem Cells: A Strategy to Improve Regeneration. Materials.

[83] Guo T., Noshin M., Baker H.B., Taskoy E., Meredith S.J., Tang Q., Ringel J.P., Lerman M.J., Chen Y., Packer J.D. (2018). 3D Printed Biofunctionalized Scaffolds for Microfracture Repair of Cartilage Defects. Biomaterials.

[84] Lu Y., Wang Y., Zhang H., Tang Z., Cui X., Li X., Liang J., Wang Q., Fan Y., Zhang X. (2021). Solubilized Cartilage ECM Facilitates the Recruitment and Chondrogenesis of Endogenous BMSCs in Collagen Scaffolds for Enhancing Microfracture Treatment. ACS Appl. Mater. Interfaces.

[85] Gao L., Beninatto R., Oláh T., Goebel L., Tao K., Roels R., Schrenker S., Glomm J., Venkatesan J.K., Schmitt G. (2023). A Photopolymerizable Biocompatible Hyaluronic Acid Hydrogel Promotes Early Articular Cartilage Repair in a Minipig Model In Vivo. Adv. Healthc. Mater..

[86] Abpeikar Z., Moradi L., Javdani M., Kargozar S., Soleimannejad M., Hasanzadeh E., Mirzaei S.A., Asadpour S. (2021). Characterization of Macroporous Polycaprolactone/Silk Fibroin/Gelatin/Ascorbic Acid Composite Scaffolds and In Vivo Results in a Rabbit Model for Meniscus Cartilage Repair. Cartilage.

[87] Yilmaz B.K., Kaga S., Kaga E., Demirel H.H., Konya M.N. (2025). Evaluation of the Efficacy of a Double-Layered and Single-Layered Synthetic Scaffold for the Treatment of Knee Osteochondral Defects—An Experimental Study. J. Orthop. Surg..

[88] Rosenzweig D.H., Carelli E., Steffen T., Jarzem P., Haglund L. (2015). 3D-Printed ABS and PLA Scaffolds for Cartilage and Nucleus Pulposus Tissue Regeneration. Int. J. Mol. Sci..

[89] Jarecki J., Waśko M.K., Widuchowski W., Tomczyk-Warunek A., Wójciak M., Sowa I., Blicharski T. (2023). Knee Cartilage Lesion Management—Current Trends in Clinical Practice. J. Clin. Med..

[90] Cook J., McCulloch P., Blazeby J., Beard D., Marinac-Dabic D., Sedrakyan A. (2013). IDEAL Framework for Surgical Innovation 3: Randomised Controlled Trials in the Assessment Stage and Evaluations in the Long Term Study Stage. Br. Med. J..

[91] Manner P.A., Tubb C.C., Levine B.R. (2018). AAOS Appropriate Use Criteria: Surgical Management of Osteoarthritis of the Knee. JAAOS-J. Am. Acad. Orthop. Surg..

[92] Solanki K., Shanmugasundaram S., Shetty N., Kim S.-J. (2021). Articular Cartilage Repair & Joint Preservation: A Review of the Current Status of Biological Approach. J. Clin. Orthop. Trauma.

[93] Bae D.K., Yoon K.H., Song S.J. (2006). Cartilage Healing After Microfracture in Osteoarthritic Knees. Arthrosc. J. Arthrosc. Relat. Surg..

[94] Goyal D., Keyhani S., Lee E.H., Hui J.H.P. (2013). Evidence-Based Status of Microfracture Technique: A Systematic Review of Level I and II Studies. Arthrosc. J. Arthrosc. Relat. Surg..

[95] Gomoll A.H. (2012). Microfracture and Augments. J. Knee Surg..

[96] Bartha L., Vajda A., Duska Z., Rahmeh H., Hangody L. (2006). Autologous Osteochondral Mosaicplasty Grafting. J. Orthop. Sports Phys. Ther..

[97] Jenko N., Ariyaratne S., Jeys L., Evans S., Iyengar K.P., Botchu R. (2024). An Evaluation of AI Generated Literature Reviews in Musculoskeletal Radiology. Surgeon.

[98] Saris D.B.F., Vanlauwe J., Victor J., Almqvist K.F., Verdonk R., Bellemans J., Luyten F.P. (2009). Treatment of Symptomatic Cartilage Defects of the Knee: Characterized Chondrocyte Implantation Results in Better Clinical Outcome at 36 Months in a Randomized Trial Compared to Microfracture. Am. J. Sports Med..

[99] Tewilliager T., Nguyen K., Ng A. (2023). Arthroscopic Cartilage Transplantation. Clin. Podiatr. Med. Surg..

[100] Clynes M.A., Harvey N.C., Curtis E.M., Fuggle N.R., Dennison E.M., Cooper C. (2020). The Epidemiology of Osteoporosis. Br. Med. Bull..

[101] Rahimzadeh P., Imani F., Faiz S.H.R., Entezary S.R., Zamanabadi M.N., Alebouyeh M.R. (2018). The Effects of Injecting Intra-Articular Platelet-Rich Plasma or Prolotherapy on Pain Score and Function in Knee Osteoarthritis. Clin. Interv. Aging.

[102] Yurtbay A., Say F., Çinka H., Ersoy A. (2022). Multiple Platelet-Rich Plasma Injections Are Superior to Single PRP Injections or Saline in Osteoarthritis of the Knee: The 2-Year Results of a Randomized, Double-Blind, Placebo-Controlled Clinical Trial. Arch. Orthop. Trauma Surg..

[103] Kumar K.H.S., Garner M., Khanduja V. (2022). An Evidence-Based Update on the Management of Articular Cartilage Defects in the Hip. J. Clin. Orthop. Trauma.

[104] Hinckel B.B., Thomas D., Vellios E.E., Hancock K.J., Calcei J.G., Sherman S.L., Eliasberg C.D., Fernandes T.L., Farr J., Lattermann C. (2021). Algorithm for Treatment of Focal Cartilage Defects of the Knee: Classic and New Procedures. Cartilage.

[105] Caldwell K.L., Wang J. (2015). Cell-based articular cartilage repair: The link between development and regeneration. Osteoarthr. Cartil..

[106] Huang B., Li P., Chen M., Peng L., Luo X., Tian G., Wang H., Wu L., Tian Q., Li H. (2022). Hydrogel Composite Scaffolds Achieve Recruitment and Chondrogenesis in Cartilage Tissue Engineering Applications. J. Nanobiotechnol..

[107] Thacher R.R., Pascual-Leone N., Rodeo S.A. (2024). Treatment of Knee Chondral Defects in Athletes. Sports Med. Arthrosc. Rev..

[108] Li L., Shi G., Wu Z., Cai Z., Wang J., Hao Z., Chen R., Piao Z., Chen C., Li J. (2025). 3D Printing of Scaffolds for Articular Cartilage/Osteochondral Regeneration: Design, Performance, and Applications. Chem. Eng. J..

[109] Thunsiri K., Pitjamit S., Pothacharoen P., Pruksakorn D., Nakkiew W., Wattanutchariya W. (2020). The 3D-Printed Bilayer’s Bioactive-Biomaterials Scaffold for Full-Thickness Articular Cartilage Defects Treatment. Materials.

[110] Ghandforoushan P., Alehosseini M., Golafshan N., Castilho M., Dolatshahi-Pirouz A., Hanaee J., Davaran S., Orive G. (2023). Injectable Hydrogels for Cartilage and Bone Tissue Regeneration: A Review. Int. J. Biol. Macromol..

[111] Zhu S., Li Y., He Z., Ji L., Zhang W., Tong Y., Luo J., Yu D., Zhang Q., Bi Q. (2022). Advanced Injectable Hydrogels for Cartilage Tissue Engineering. Front. Bioeng. Biotechnol..

[112] Mistry H., Connock M., Pink J., Shyangdan D., Clar C., Royle P., Court R., Biant L.C., Metcalfe A., Waugh N. (2017). Autologous Chondrocyte Implantation in the Knee: Systematic Review and Economic Evaluation. Health Technol. Assess. Winch. Engl..

[113] Krill M., Early N., Everhart J.S., Flanigan D.C. (2018). Autologous Chondrocyte Implantation (ACI) for Knee Cartilage Defects: A Review of Indications, Technique, and Outcomes. JBJS Rev..

[114] Basad E., Ishaque B., Bachmann G., Stürz H., Steinmeyer J. (2010). Matrix-Induced Autologous Chondrocyte Implantation versus Microfracture in the Treatment of Cartilage Defects of the Knee: A 2-Year Randomised Study. Knee Surg. Sports Traumatol. Arthrosc..

[115] Nassar J.E., Guerin G., Keel T., Russo R., Familiari F., Tollefson L.V., LaPrade R.F. (2025). Autologous Chondrocyte Implantation, Matrix-induced Autologous Chondrocyte Implantation, Osteochondral Autograft Transplantation and Osteochondral Allograft Improve Knee Function and Pain with Considerations for Patient and Cartilage Defects Characteristics: A Systematic Review and Meta-analysis. Knee Surg. Sports Traumatol. Arthrosc..

[116] Anders S., Volz M., Frick H., Gellissen J. (2013). A Randomized, Controlled Trial Comparing Autologous Matrix-Induced Chondrogenesis (AMIC^®^) to Microfracture: Analysis of 1- and 2-Year Follow-Up Data of 2 Centers. Open Orthop. J..

[117] Hangody L., Füles P. (2003). Autologous Osteochondral Mosaicplasty for the Treatment of Full-Thickness Defects of Weight-Bearing Joints: Ten Years of Experimental and Clinical Experience. J. Bone Jt. Surg..

[118] Bodenbeck E.-M., Böpple J.C., Doll J., Bürkle F., Schmidmaier G., Fischer C. (2024). Earlier Consolidation and Improved Knee Function of Medial Open Wedge High Tibial Osteotomy with Autologous Bone Graft. Eur. J. Orthop. Surg. Traumatol..

[119] Goulian A.J., Goldstein B., Saad M.A. (2025). Advancements in Regenerative Therapies for Orthopedics: A Comprehensive Review of Platelet-Rich Plasma, Mesenchymal Stem Cells, Peptide Therapies, and Biomimetic Applications. J. Clin. Med..

[120] Guo X., Ma Y., Min Y., Sun J., Shi X., Gao G., Sun L., Wang J. (2023). Progress and Prospect of Technical and Regulatory Challenges on Tissue-Engineered Cartilage as Therapeutic Combination Product. Bioact. Mater..

[121] Wang L., Guo X., Chen J., Zhen Z., Cao B., Wan W., Dou Y., Pan H., Xu F., Zhang Z. (2022). Key Considerations on the Development of Biodegradable Biomaterials for Clinical Translation of Medical Devices: With Cartilage Repair Products as an Example. Bioact. Mater..

[122] Belsky K. (2021). Current state of U.S. Food and Drug Administration regulation for human cell, tissue, and cellular and tissue-based products: Minimal manipulation and homologous use criteria. J. Bone & Cartilage Res..

[123] Tor J.Q., Le Q.B., Ezhilarasu H., Chan W.W., Choudhury D. (2025). Advancements and Regulations of Biomanufacturing Cell-Based Cartilage Repair Therapies. Trends Biotechnol..

[124] Moran C.J., Ramesh A., Brama P.A.J., O’Byrne J.M., O’Brien F.J., Levingstone T.J. (2016). The Benefits and Limitations of Animal Models for Translational Research in Cartilage Repair. J. Exp. Orthop..

[125] Nordberg R.C., Otarola G.A., Wang D., Hu J.C., Athanasiou K.A. (2022). Navigating Regulatory Pathways for Translation of Biologic Cartilage Repair Products. Sci. Transl. Med..

[126] Nordberg R.C., Bielajew B.J., Takahashi T., Dai S., Hu J.C., Athanasiou K.A. (2024). Recent Advancements in Cartilage Tissue Engineering Innovation and Translation. Nat. Rev. Rheumatol..

[127] Gracitelli G.C., Meric G., Pulido P.A., McCauley J.C., Bugbee W.D. (2015). Osteochondral Allograft Transplantation for Knee Lesions after Failure of Cartilage Repair Surgery. Cartilage.

[128] Kwon H., Brown W.E., Lee C.A., Wang D., Paschos N., Hu J.C., Athanasiou K.A. (2019). Surgical and Tissue Engineering Strategies for Articular Cartilage and Meniscus Repair. Nat. Rev. Rheumatol..

[129] Piñeiro-Ramil M., Gómez-Seoane I., Rodríguez-Cendal A.I., Fuentes-Boquete I., Díaz-Prado S. (2025). Mesenchymal Stromal Cells-Derived Extracellular Vesicles in Cartilage Regeneration: Potential and Limitations. Stem Cell Res. Ther..

[130] Liu Y.-B., Liu X., Li X.-F., Qiao L., Wang H.-L., Dong Y.-F., Zhang F., Liu Y., Liu H.-Y., Ji M.-L. (2025). Multifunctional Piezoelectric Hydrogels under Ultrasound Stimulation Boost Chondrogenesis by Recruiting Autologous Stem Cells and Activating the Ca^2+^/CaM/CaN Signaling Pathway. Bioact. Mater..

[131] Ricotti L., Cafarelli A., Manferdini C., Trucco D., Vannozzi L., Gabusi E., Fontana F., Dolzani P., Saleh Y., Lenzi E. (2024). Ultrasound Stimulation of Piezoelectric Nanocomposite Hydrogels Boosts Chondrogenic Differentiation in Vitro, in Both a Normal and Inflammatory Milieu. ACS Nano.

[132] Lin J., Li S., Peng Y., Qiu C., Yang M., Guo J., Yu B., Chen Y. (2025). Ultrasound-Triggered Injectable Piezoelectric Nanocomposite Hyaluronic Acid-Based Hydrogel for Modulating Inflammation and Chondrogenesis in Osteoarthritis Treatment. Carbohydr. Polym..

[133] Liu D., Wang X., Gao C., Zhang Z., Wang Q., Pei Y., Wang H., Tang Y., Li K., Yu Y. (2024). Biodegradable Piezoelectric-Conductive Integrated Hydrogel Scaffold for Repair of Osteochondral Defects. Adv. Mater..

[134] Liang J., Huang X., Qin K., Wei H., Yang J., Liu B., Fan Z. (2025). Implanted Magnetoelectric Bionic Cartilage Hydrogel. Adv. Mater..

